# CXCR3 chemokine receptor guides *Trypanosoma cruzi*-specific T-cells triggered by DNA/adenovirus ASP2 vaccine to heart tissue after challenge

**DOI:** 10.1371/journal.pntd.0007597

**Published:** 2019-07-29

**Authors:** Camila Pontes Ferreira, Leonardo Moro Cariste, Barbara Ferri Moraschi, Bianca Ferrarini Zanetti, Sang Won Han, Daniel Araki Ribeiro, Alexandre Vieira Machado, Joseli Lannes-Vieira, Ricardo Tostes Gazzinelli, José Ronnie Carvalho Vasconcelos

**Affiliations:** 1 Department of Microbiology, Immunology and Parasitology, Federal University of São Paulo, São Paulo, Brazil; 2 Department of Biosciences, Federal University of São Paulo, Santos, Brazil; 3 Department of Biophysics, Federal University of São Paulo, São Paulo, Brazil; 4 René Rachou Research Center, Fiocruz, Minas Gerais, Brazil; 5 Laboratory of Biology of the Interactions, Oswaldo Cruz Institute/Fiocruz, Rio de Janeiro, Brazil; 6 Division of Infectious Diseases and Immunology, University of Massachusetts Medical School, Worcester, United States ofAmerica; University of Georgia, UNITED STATES

## Abstract

CD8^+^ T lymphocytes play an important role in controlling infections by intracellular pathogens. Chemokines and their receptors are crucial for the migration of CD8^+^ T-lymphocytes, which are the main IFNγ producers and cytotoxic effectors cells. Although the participation of chemokine ligands and receptors has been largely explored in viral infection, much less is known in infection by *Trypanosoma cruzi*, the causative agent of Chagas disease. After *T*. *cruzi* infection, CXCR3 chemokine receptor is highly expressed on the surface of CD8^+^ T-lymphocytes. Here, we hypothesized that CXCR3 is a key molecule for migration of parasite-specific CD8^+^ T-cells towards infected tissues, where they may play their effector activities. Using a model of induction of resistance to highly susceptible A/Sn mice using an ASP2-carrying DNA/adenovirus prime-boost strategy, we showed that CXCR3 expression was upregulated on CD8^+^ T-cells, which selectively migrated towards its ligands CXCL9 and CXCL10. Anti-CXCR3 administration reversed the vaccine-induced resistance to *T*. *cruzi* infection in a way associated with hampered cytotoxic activity and increased proapoptotic markers on the H2K^K^-restricted TEWETGQI-specific CD8^+^ T-cells. Furthermore, CXCR3 receptor critically guided TEWETGQI-specific effector CD8^+^ T-cells to the infected heart tissue that express CXCL9 and CXCL10. Overall, our study pointed CXCR3 and its ligands as key molecules to drive *T*. *cruzi*-specific effector CD8^+^ T-cells into the infected heart tissue. The unveiling of the process driving cell migration and colonization of infected tissues by pathogen-specific effector T-cells is a crucial requirement to the development of vaccine strategies.

## Author summary

Chemokine receptors and cell adhesion molecules are essential for T lymphocytes migration into infected tissues. Previously, our group demonstrated that CXCR3 receptor was highly expressed on specific CD8^+^ T-cells surface after immunization and infection by *T*. *cruzi*. Also, recirculation of specific CD8^+^ T-cells was more important than proliferation to control the infection by *T cruzi*. As CD8^+^ T lymphocytes are responsible for controlling *T*. *cruzi* infection by releasing IFN-γ or by direct cytotoxicity against infected target cells, our aim was to analyze the role of the chemokine receptor CXCR3 in the migration of specific CD8^+^ T-cells towards infected tissues. Our results revealed that intervention on CXCR3 by administration of a blocking anti-CXCR3 antibody decreased CD8^+^ T-cell migration, hampering the access of parasite-specific effector cell into the heart tissue of mice infected by *T*. *cruzi*. Therefore, to induce the appropriate migration footmarks is crucial for drive the pathogen-specific effector to sites of infection and, therefore, to clarify this requirement is a crucial strategy for vaccine development.

## Introduction

The causative agent of Chagas disease *Trypanosoma cruzi* is an intracellular parasite that infects a variety of cells of the mammalian host [1,2]. The activation of adaptive immune response occurs by recruiting T lymphocytes to the infection sites after the presentation of trypomastigote/amastigote-related proteins via MHC-I or MHC-II [3,4]. CD8^+^ T lymphocytes are the cells primarily responsible for controlling intracellular pathogens such as *T*. *cruzi* [5–7]. Their relevance to the control of *T*. *cruzi* infection was demonstrated during the infection of CD8-deficient mice, or by the blockade of this molecule using monoclonal antibodies; in both cases, animals did not survive to infection [8]. The multiple antiparasitic mechanisms mediated by these cells include secretion of cytokines and direct cytotoxicity against infected cells [9,10].

The importance of the immune response mediated by CD8^+^ T lymphocytes, which promote resistance to *T*. *cruzi* infection, has led several groups to investigate different vaccine strategies [11]. Our group has been studying the prime-boost protocol that uses plasmid vector for priming and a replication-defective human adenovirus type 5 (AdHu5 vector) [9,12] for boosting, both containing an insertion of the amastigote surface protein 2 (ASP2) gene ASP2. That immunization protocol can induce a strong CD8-mediated response able to protect the highly susceptible A/Sn mice to experimental *T*. *cruzi* infection [13,14]. Recently, we have shown that more than proliferative response, the specific CD8^+^ T-cells need to recirculate to exert protection against infection in A/Sn mice [9,13].

Chemokine molecules are small chemotactic molecules responsible for the guidance of leukocyte migration during homeostasis and inflammation [15]. In addition to cell migration, chemokines acting as costimulatory molecules involved in T-lymphocytes activation, differentiation and proliferation [16,17]. Pro-inflammatory chemokines are induced by infection with different pathogens and molecular inflammatory stimuli [18]. Chemokines expression are induced by an IFN-γ- and TNF-enriched Th1-type immune response triggered by infection with intracellular pathogens [19,20] such as *T*. *cruzi* [21–23]. Naive T cells differentiate into cytokine-producing cells such as IFN-γ, IL-2 and TNF; this differentiation occurs through the expression of interleukin IL-12 and the T-bet transcription factor [24].

Differentiated effector T cells express high levels of the CXC-chemokine receptor CXCR3, whereas its ligands CXCL10 (IP-10), CXCL11, and CXCL9 (MIG) are produced by antigen presenting cells present in the infected tissues [25]. The role of CXCR3 receptor and the migration of effector T lymphocytes during Th1 type responses have already been demonstrated in a murine model infected by the protozoan *Toxoplasma gondii*. This receptor was highly expressed on CD4^+^ T cells and was responsible for the migration of T lymphocytes to the intestine, enabling the control of parasite load and tissue damage, and consequently the survival of infected mice [26]. In addition to cell migration, chemokine receptors such as CXCR3 affect the differentiation of T lymphocytes. Indeed, recently published studies have shown that the absence of CXCR3 favors the differentiation of memory effector CD8^+^ T cells [27,28].

Considering that CXCL10 and CXCL9 are expressed in heart tissue of acute and chronically *T*. *cruzi*-infected mice presenting a CD8^+^ T-cell-enriched myocarditis [21,29], here we hypothesized that CXCR3 is a key molecule for migration of specific CD8^+^ T-cells towards infected tissues. Using a model of prime-boost immunization in highly susceptible to *T*. *cruzi* infection A/Sn mice, we analyzed the role of CXCR3 receptor present on pathogen-specific CD8^+^ T-cells migration, compartmentalization and effector functions. Further, we used an anti-CXCR3 blocking antibody as a tool to interfere in the migration process of CD8^+^ T-cells and analyzed susceptibility to infection, migration pattern, tissue colonization and effector activity. Therefore, we aimed to shed light on the importance of CXCR3-driven cell migration, and its role to protection and tissue injury in *T*. *cruzi* infected hosts. This knowledge may contribute to the strategies of vaccine development against intracellular pathogens.

## Methods

### Ethics statement and mice

This study was carried out in strict accordance with the recommendations in the Guide for the Care and Use of Laboratory Animals of the Brazilian National Council of Animal Experimentation (http://www.cobea.org.br). The protocol was approved by the Ethical Committee for Animal Experimentation at the Federal University of Sao Paulo (Id # CEP 7559051115). Eight-weeks-old female mice, A/Sn, C57BL/6 or CD8-deficient mice (*cd8*^-/-^), were purchased from the Federal University of São Paulo (CEDEME). CCR2 deficient mice (*ccr2*^-/-^) were kindly supplied by Dr. João Santana, Ribeirão Preto School of Medicine-FMPR. Blood trypomastigotes of Y strain of *T*. *cruzi* were maintained by weekly passages in A/Sn mice at the Xenodiagnosis Laboratory of Dante Pazzenese Cardiology Institute. For *in vivo* experiments, each mouse was inoculated with 150 (A/Sn) or 10^4^ trypomastigotes forms from blood (C57BL/6) diluted in 0.2 mL PBS and administered subcutaneously (s.c.) at the base of the tail. Parasitemia was determined by the examination of 5 μL of blood, and parasites were counted with a light microscope.

### Immunization protocol

Heterologous prime-boost immunization protocol with plasmid pIgSPCl.9 and the human replication-defective adenovirus type 5 containing the ASP2 gene, as described previously [12,30], was used. Briefly, the immunization consists of a dose of plasmid DNA (100 μg) as a prime (pcDNA3 control or pIgSPClone9) and three weeks later, mice were boosted with 2x10^8^ plaque-forming units (pfu) of the adenoviral vectors Adβ-gal or AdASP2. Both injections were performed via intramuscular route (*Tibialis anterior* muscle). After 15 days of boosting mice were infected with *T*. *cruzi*.

### Treatments

Blocking monoclonal antibodies anti-CXCR3 (clone CXCR3-173, BioXcell), anti-CCL2 (clone 2H5, BioXcell) and isotype control antibody Rat IgG2a (clone 2A3, BioXcell), were administered via i.p route on the same day of infection and every 48 hours until 20 days after infection. The quantity of antibodies administered was 250 μg of mAb/mouse. The dose was the same used in previously studies [31]. The groups were divided as shown below:

1. βgal+*T*.*cruzi*: Immunized with controls plasmid pcDNA3/Adβgal and challenged with *T*.*cruzi*;
2. ASP2+*T*.*cruzi*: Immunized with pIgSPclone9/AdASP2, challenged with *T*.*cruzi* and treated with isotype control antibody.
3. ASP2+αCXCR3+*T*.*cruzi*: Immunized with pIgSPclone9/AdASP2, challenged with *T*. *cruzi* and treated with anti-CXCR3 antibody.

### Peptide

TEWETGQI immunodominant peptide described earlier [32], was synthesized by GenScript (Piscataway, New Jersey) and used for *in vitro* and *ex vivo* stimulation of splenocytes. The multimer H2K^K-^TEWETGQI (Immudex Copenhagen, Denmark) was used for specific CD8^+^ T cell detection.

### Quantification of parasite burden

Hearts and spleens from βgal+*T*.*cruzi*, ASP2+*T*.*cru*zi and ASP2+αCXCR3+*T*.*cruzi* were used for DNA extraction. The DNA extraction, the specific primers for a satellite DNA region of the parasite (*T*. *cruzi*) and the qPCR reaction using TaqMan Universal Master Mix II with UNG were adapted from Piron and colleagues [33].

### ELISpot

Briefly, 1x10^6^ responder cells from spleen were incubated with 3x10^5^ antigen-presenting cells in complete medium (1% NEAA, 1% L-glutamine, 1% vitamins and 1% pyruvate, 0,1% 2-ME, 10% FBS (HyClone), 20 U/mL mouse recombinant IL-2 (SIGMA) and on a plate previously coated with capture antibody those cells were incubated in the presence or absence of 10 μM of peptide TEWETGQI. After 24 hours the plates were washed with PBS and PBS-Tween 20 (0.05% Tween). Each well received 2 μg/mL of biotinylated anti-mouse monoclonal antibody (clone XMG1.2, Pharmingen). The plates were incubated with streptavidin-peroxidase (BD) and developed by adding peroxidase substrate (50mM Tris-HCl, pH 7.5, containing 1 mg/ml DAB and 1 μL/ml 30% hydrogen peroxide, both from SIGMA). The number of IFN-γ-producing cells was determined using a stereoscope.

### Intracellular cytokine staining and flow cytometry

Two million cells from spleen were treated with ACK buffer (NH_4_Cl, 0.15 M; KHCO_3_, 10 mM; Na_2_-EDTA 0.1 mM; pH = 7.4). Both spleen and heart cells were stained with H2K^k^-TEWETGQI multimer for 10 minutes at RT. The splenocytes cell surface was stained for 30 min at 4°C. The following antibodies were used for surface staining: anti-CD3 APCcy7 (clone 145-2C11, BD), anti-CD8 PERCP or anti-CD8 PACIFIC BLUE (clone 53–67, BD), anti-CD11a FITC (clone 2D7, BD), anti-CD11c APCcy7 (clone HL3, BD), anti-CD44 FITC (clone IM7, BD), anti-CD62L PE (clone MEL-14, BD), anti-CXCR3 PERCP/Cy5.5 (clone 173, BioLegend), anti-CD27 FITC (clone LG3A10, BD), anti-CD4 PEcy7 (clone RM4-5, BD) anti-KLRG1 FITC (clone 2F1, eBioscience), anti-CD49d PEcy7 (clone R1-2, BD), anti-CD69 PERCP (clone H1.2F3, BD), anti-CD43 PEcy7 (1B11, BioLegend), anti-CD95 PEcy7 (clone JO2, BD), anti-CD25 FITC (clone LG3A10, BD), anti-CD127 PE (clone SB/199, BD), anti-CD122 FITC (clone TM-β1, BD), anti-CD38 PE (clone 90, BD), anti-β7 PERCP (clone FIB27, BioLegend), anti-CD31 FITC (clone MEC 13.3, BD), anti-CD272 PE (clone 8F4, eBioscience), anti-PD-1 FITC (clone J43, eBioscience), anti-CTLA-4 PE (clone UC10-4B9, eBioscience), and anti-CCR7 PE (clone 4B12, BD). For annexin V and 7-AAD assays, 2x10^6^ of spleen cells were labeled with multimer, subsequently, the cells were stained according to the Annexin-PE Kit protocol (BD Pharmingen).

To detect IFN-γ, TNF or granzyme B by intracellular staining, 2x10^6^ cells/mL in cell culture medium containing CD107a FITC antibody (clone 1D4B, BD), anti-CD28 (clone 37.51, BD), BD Golgi-Plug (1 μL/mL) and monensin (5 μg/mL) were incubated in presence or absence of 10 μM of peptide TEWETGQI, no longer than 12 hours in V-bottom 96-well plates with a final volume of 200 μL at 37°C and containing 5% CO_2_. Cells were washed and stained for surface markers with anti-CD8 PERCP antibody (clone 53–6.7, BD) on ice for 30 min. Cells were then double washed in buffer containing PBS, 0.5% BSA, and 2 mM EDTA, fixed and permeabilized with BD perm/wash buffer. After the double wash procedure with BD perm/wash buffer, cells were stained for intracellular markers using APC-labeled anti-IFN-γ (clone XMG1.2, BD), PE-labeled anti-TNF (clone MP6-XT22, BD) and PE-labeled anti-granzyme B (clone GB11, Invitrogen) for 20 minutes on ice. Finally, cells were washed twice with BD perm/wash buffer and fixed in 1% PBS-paraformaldehyde. At least 700,000 cells were acquired on a BD FACS Canto II flow cytometer and then analyzed with FlowJo.

### Purification of heart lymphocytes

For isolating lymphocytes in the heart, we used the protocol described by Gutierrez and colleagues [34]. Briefly, hearts collected from 5 mice at day 20 dpi were minced, pooled, and incubated for 1h at 37°C with RPMI 1640, supplemented with NaHCO_3_, penicillin-streptomycin gentamicin, and 0.05 g/mL of liberase blendzyme CI (Roche, Basel, Switzerland). The tissues were processed in Medimachine (BD Biosciences) with phosphate buffered saline (PBS) containing 0.01% bovine serum albumin (BSA). After tissue digestion and washes the lymphocytes were isolated by Ficoll gradient (Sigma).

### *In vivo* BrdU incorporation

On the same day of infection mice were treated with 2 mg of BrdU (5-bromo-2'-deoxiuridine, SIGMA) via i.p route and every 48 hours, until 20 days after challenge. Then, 2x10^6^ splenocytes were stained with H2K^k^-TEWETGQI multimer; BrdU staining was performed according to the BrdU-FITC Kit protocol (BD Pharmingen). At least 700,000 cells were acquired on a BD FACS Canto II flow cytometer and analyzed with FlowJo 8.7.

### *In vitro* proliferation

Splenocytes from βgal+*T*.*cruzi*, ASP2+*T*.*cruzi*, and ASP2+αCXCR3+*T*.*cruzi* were stained with 1,25 μM of carboxyfluorescein diacetate succinimidyl diester (CFSE; Molecular Probes, Eugene, OR, USA), stimulated with TEWETGQI peptide, and incubated during 6 days at 37°C. Following, splenocytes were stained with H2K^k^-TEWETGQI multimer and the percentage of CFSE dilution was analyzed. At least 700,000 cells were acquired on a BD FACS Canto II flow cytometer and analyzed with FlowJo 8.7.

### *In vivo* cytotoxicity assay

We used the protocol described by Silverio et al [10]. Briefly, splenocytes collected from naive A/Sn mice were treated with ACK buffer to lyse the red blood cells. Those cells were divided into two populations and were labeled with the fluorogenic dye CFSE (Molecular Probes, Eugene, OR, USA) at a final concentration of 10 μM (CFSE^high^) or 1 μM (CFSE^low^). CFSE^high^ cells were coated with 2.5 μM of the TEWETGQI ASP2 peptide for 40 minutes at 37°C. CFSE^low^ cells remained uncoated. Subsequently, CFSE^high^ cells were washed and mixed with equal numbers of CFSE^low^ cells before intravenous injection (2x10^7^ cells per mouse) into recipient mice. Spleen cells from the recipient mice were collected at 20 hours after adoptive cell transfer and fixed with 1.0% paraformaldehyde. At least 100,000 cells were acquired on a BD FACS Canto II flow cytometer and analyzed with FlowJo 8.7. The percentage of specific lysis was determined using the following formula:

$$\%lysis=\frac{1-\left(\%CFSE^{high\;}infected/\%CFSE^{low}\;infected\right)}{\left(\%CFSE^{high}\;naive/\%CFSE^{low}\;naive\right)\;x\;100.}$$

### Quantification of chemokine genes by RT-PCR

Total RNA from hearts of naïve, βgal+*T*.*cruzi*, ASP2+*T*.*cruzi* and ASP2+αCXCR3+*T*.*cruzi* groups, was extracted by using TRIzol and complementary DNA prepared using SuperScript IV VILO (Applied Biosystems). Quantitative PCR was performed with TaqMan Universal Master Mix II (Applied Biosystems) using a StepOne thermocycler (Applied Biosystems). We used a customized plate—TaqMan Array 96-well Mouse Chemokines Plate targets genes.

### Histology and immunohistochemistry analysis

Hearts were fixed in 10% formalin, and then dehydrated, embedded in paraffin blocks, and sectioned in microtome. Staining was obtained with hematoxylin and eosin, and the number of amastigotes nests was quantified using a light microscope with 40x objective lens. Overall, 25 fields/group were counted. For immunohistochemistry the mice’s hearts were removed and frozen in Tissue-Tek O.C.T (Sakura Fineteck). The blocks were sectioned in cryostat (7 μm thickness) and then fixed in ice-cold acetone for 15 minutes. The samples were stained with 20 μg of the biotinylated anti-CD8 antibody (clone 53–6.7, RD systems) for 12 hours. After incubation, samples were labeled with streptavidin Alexa Fluor 488 (Thermo Fischer) at the concentration of 0.5 mg/mL, diluted 1:100 for 1 hour at room temperature. The DAPI (4',6-diamidino-2-phenylindole, Sigma) dye was used for labeling the cell nucleus, the concentration used was 5 mg/mL. The images were acquired in Confocal Leica TCS SP8 CARS microscope from Institute of Pharmacology and Molecular Biology (INFAR). The images were obtained using the 63x objective and processed in ImageJ software.

### Luminex assay

Mice’s serum was collected on days 0, 6, 8, 10, 12 and 14 after infection in order to quantify the ligands of CXCR3, IP-10 (CXCL10), MIG (CXCL9), also, MCP-1 (CCL2) and RANTES (CCL5). The quantification was performed according to the MCYTOMAG-70K Kit protocol (Merck Millipore). Luminex xMAP in the Institute of Pharmacology and Molecular Biology (INFAR) was used to read the plates.

### Adoptive transfer to CD8 deficient mice

CD8^+^T cells from spleens of βgal+*T*.*cruzi*, ASP2+*T*.*cruzi*, and ASP2+αCXCR3+*T*.*cruzi* groups were purified using CD8a^+^ T cell isolation kit (Miltenyi) and labeled with 10 μM of CFSE. 1x10^6^ CD8^+^ T cells were transferred via i.v. route to infected CD8 deficient mice (day 6 of infection) and 6 days afterwards, the number of CD8^+^ CFSE cells on recipient mice’s hearts were analyzed by fluorescent microscopy.

### Migration index

CD8^+^ T-cells from mice were purified using a negative selection kit (Miltenyi Biotec). A transwell microplate (Corning) with 5μm membrane pore was used to carry out the migration assay. For each condition tested, lower chambers of transwell were filled with 600 μL in the absence or presence of 100ng/mL CXCL9, CXC10 and/or CXCL11 (RD systems). CD8^+^ T-cells (5x10^4^ in 300 μL) were deposited in the upper chamber of transwell and incubated for 3h at 37°C. CD8^+^ T-cells were harvested from the lower chamber and counted using cytometer. The migration index was calculated through the ratio of cells that migrated in the presence of medium and ligands.

### Statistical analysis

Parasitemia, number of IFN-γ-producing cells (ELISpot), and absolute number of CD8^+^ T-cells were compared by analysis of unidirectional variance (ANOVA); subsequently, the Tukey’s HSD test was used (http://vassarstats.net/). The survival rate was compare using the Log-rank test using GraphPad Prism 7. The expression of molecules was compared using MFI (Mean Fluorescence Intensity), and the *naive* group MFI was taken as the baseline. MFI was determined by the FlowJo software (version 9.9). The Kaplan-Meier method was employed to compare survival rates of the studied groups. All statistical tests were performed with GraphPad Prism 5.0 (La Jolla, CA, USA). Differences were considered statistically significant when *P* < 0.05.

## Results

### CXCR3 receptor is highly expressed on T lymphocyte surface after immunization and *T*. *cruzi* infection

**Fig 1:**
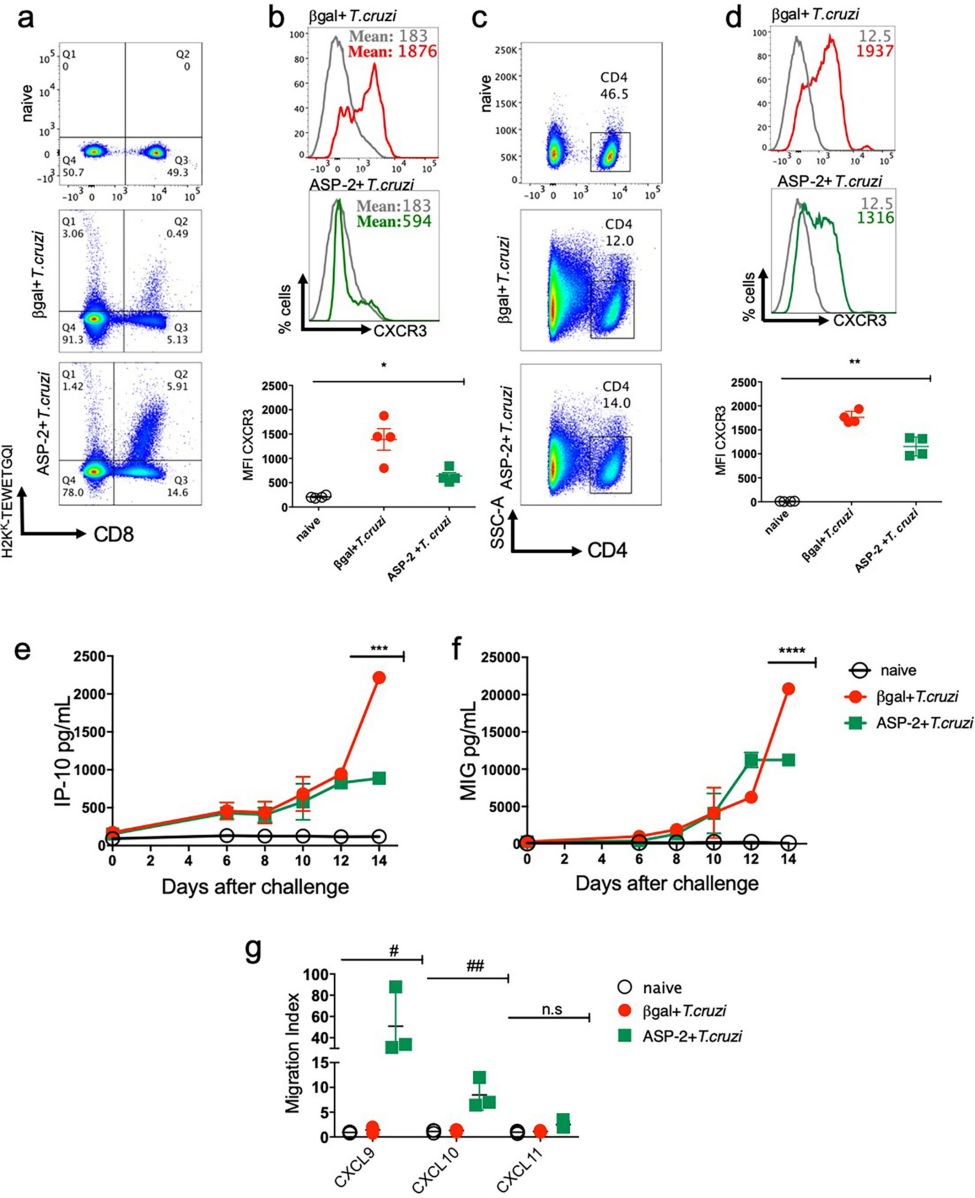
Specific CD8^+^ and activated CD4^+^ T-cells expressed CXCR3 receptor and CXCR3 ligands in the serum of *T*. *cruzi* infected mice. A/Sn mice were immunized with heterologous prime-boost protocol as described in the methods section. Thirty-six days after the first immunization, mice were challenged with 150 blood trypomastigotes forms of Y strain of *T*. *cruzi*. The experimental groups are mice immunized with a control vector and infected (βgal+*T*. *cruzi*) and mice immunized with ASP2 gene and infected (ASP2+*T*.*cruzi*). a-The dot-plot graphs represent the frequency of specific H2K^k^-TEWETGQI CD8^+^ T cells in the spleen of βgal+*T*.*cruzi* and ASP2+*T*.*cruzi* groups. b-The histograms and graph indicate the MFI (mean fluorescence intensity) of CXCR3 expression on specific H2K^K^-TEWETGQI CD8^+^ T cells surface. Each line corresponds to a group: naïve (grey), βgal+*T*.*cruzi* (red) or ASP2+*T*.*cruzi* (green). c-Dot-plots graphs show the frequency of CD4^+^ T cells gated as CD44^high^ and CD62L^low^ population. d-The histograms and graph indicate the MFI (mean fluorescence intensity) of CXCR3 expression on activated CD4^+^ T cells surface. Each line corresponds to a group: naïve (grey), βgal+*T*.*cruzi* (red) or ASP2+*T*.*cruzi* (green) groups. e-Quantity of IP-10 (CXCL10) and (f) MIG (CXCL9) chemokines in pg/mL in serum of naïve, βgal+*T*.*cruzi* and ASP2+*T*.*cruzi* groups. Chemokines were measured on days zero, 6, 8,10, 12 and 14 after infection, but the statistical analysis was performed only on day 14. g-Graph represents the migration index of CD8^+^ T cells from the spleen of naïve, βgal+*T*.*cruzi* and ASP2+*T*.*cruzi* groups after stimulation with CXCL9, CXCL10, and CXCL11 chemokines. Results are shown as individual values and the mean ± SEM for each group (n = 4). One of two independent experiments is presented. Statistical analysis was performed using the One-Way ANOVA. Symbols indicate that the values observed were significantly different between the groups (*p = 0,0005; **p = 0,0001; ***p<0,0001; ****p<0,0001; ^#^p<0,05; ^##^p<0,01) and n.s means no significant.

To investigate whether T lymphocytes expressed CXCR3 receptor on T cells surface after immunization and/or infection with *T*. *cruz*i, splenic parasite antigen-specific CD8^+^ and activated CD4^+^ T-cells of A/Sn mice were labeled on day 20 after infection. The dot-plot graphs show the frequency of specific CD8^+^ T cells, gated as positive for H2K^K^-TEWETGQI (Fig 1A). In infected group, immunized with the control DNA/adenovirus encoding the βgal (βgal+*T*.*cruzi* group), the frequency of H2K^K^-restricted TEWETGQI-specific CD8^+^ T-cells was lower (Q2: 0.49%) when compared to that found in mice immunized with DNA/adenovirus encoding the ASP-2 (Q2: 5.91%) and further infected with *T*. *cruzi* (ASP2+*T*.*cruzi*), as shown in Fig 1A. Also, during the infection, the Mean of Fluorescence Intensity (MFI) of CXCR3 receptor was higher in βgal+*T*.*cruzi* than in ASP2+*T*.*cruzi* group (Fig 1B). When CD44^high^ and CD62L^low^ activated CD4^*+*^ T-cells were gated (Fig 1C), we observed that differently in specific CD8^+^ T cells, CXCR3 expression was higher expressed and similar in both experimental groups (βgal+*T*.*cruzi* and ASP2+*T*.*cruzi*) (Fig 1D). In addition to enhanced CXCR3 expression, we evaluated the concentrations of the CXCR3 ligands IP-10/CXCL10 and MIG/CXCL9 in the serum of mice at days 0, 6, 8, 10, 12, and 14 after infection. As shown in Fig 1E and 1F, the concentrations of CXCL9 and CXCL10 increased on day 10 after challenge in both groups, βgal+*T*.*cruzi* and ASP2+*T*.*cruzi* and reaching the maximum concentration on day 14 after infection. Importantly, the levels of IP-10 and MIG were higher in the serum of mice of the group βgal+*T*.*cruzi* when compared to the ASP2+*T*.*cruzi* group. We also measured the levels of the chemokines CCL2/MCP-1 and CCL5/RANTES, but no differences were observed in those chemokine levels when compared to those found in the serum of naïve group (S1A and S1B Fig). Interestingly, purified CD8^+^ T cells from spleen of ASP2+*T*.*cruzi* mice group had higher migration index after chemotaxis induced by the recombinant proteins CXCL9 and CXCL10, when compared to cells harvested from mice of βgal+*T*.*cruzi* group (Fig 1G). No significant migration was detected under stimulation with CXCL11, supporting the selective effect of CXCL9 and CXCL10 to induce *ex vivo* chemotaxis of CD8^+^ T-cells. These results showed that CXCR3 is highly expressed on T cell surface as well as CXCR3 ligands (CXCL9 and CXCL10), especially in the infected group; however, the immunized group’s CD8^+^ T cells showed more migration capacity after stimulation with CXCL9 and CXCL10 recombinant proteins.

### CXCR3, but not CCR2 chemokine receptor is important to survival of A/Sn mice challenged with *T*. *cruzi*

**Fig 2:**
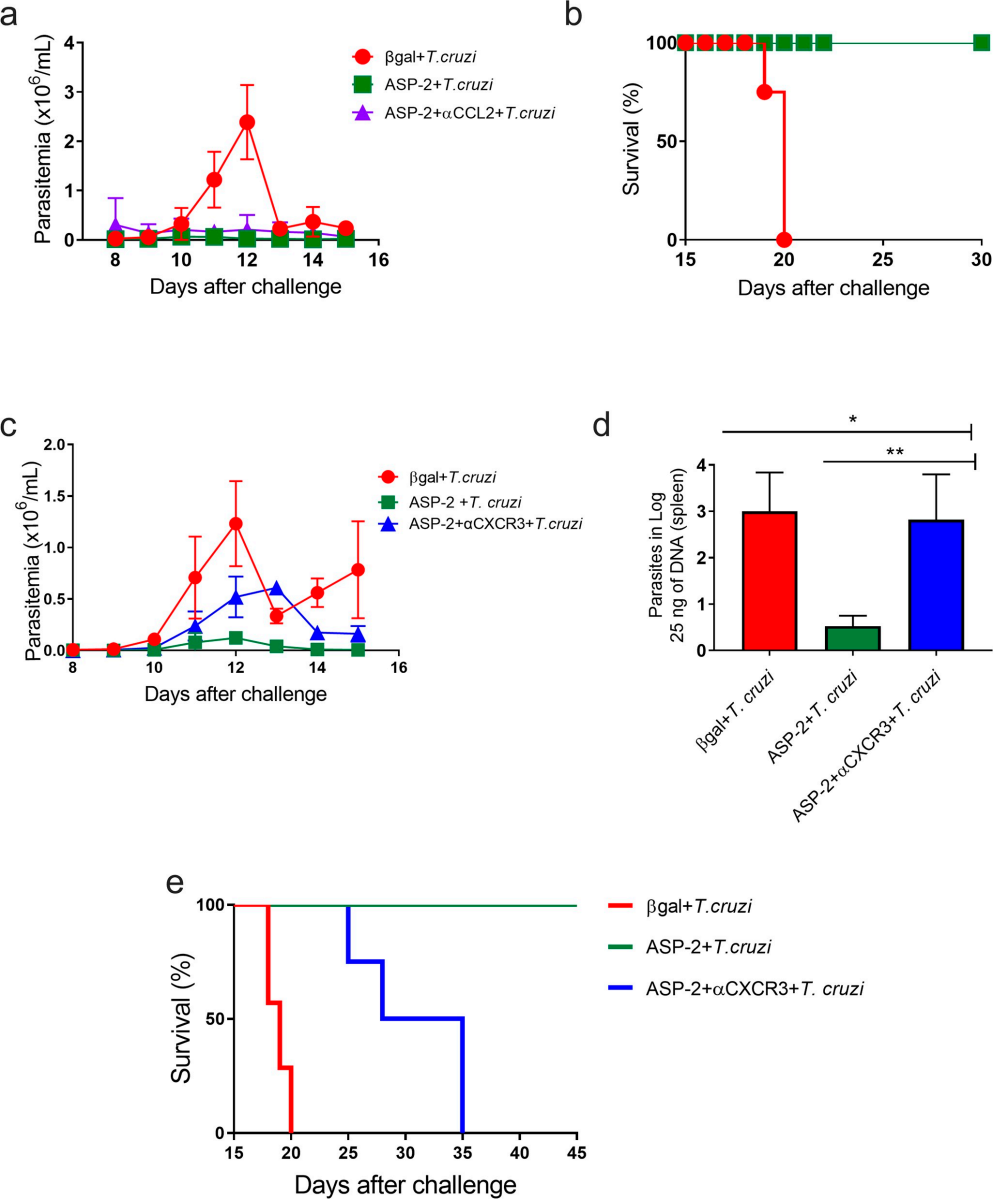
Parasitemia and survival of mice treated with anti-CCL2 or anti-CXCR3 antibody. A/Sn mice were immunized with the heterologous prime-boost protocol described earlier, and 15 days after the last dose of immunization, mice were challenged with 150 blood forms of Y strain of *T*. *cruzi* and treated, on the same day, with anti-CCL2 or anti-CXCR3 monoclonal antibodies. a-Linear parasitemia scale of βgal+*T*.*cruzi* (red), ASP2+*T*.*cruzi* (green) and ASP2+αCCL2+*T*.*cruzi* (purple) groups. In A/Sn mice, the peak of parasitemia was on day 12 after infection. b-Survival rate curve of mice was followed up until 30 days of infection. Kaplan–Meier curves for survival of the different groups were compared using the Log-Rank test (all groups p<0,002; groups ASP2+*T*.*cruzi* and ASP2+αCCL2+*T*.*cruzi* p>0,9). c-Linear parasitemia scale of βgal+*T*.*cruzi* (red), ASP2+*T*.*cruzi* (green) and ASP2+αCXCR3+*T*.*cruzi* (blue) groups. d-Number of parasites in the spleen, measured by Real time qPCR. Asterisks indicate that the values were statistically different after analysis using One-Way ANOVA and Tukey’s HSD tests (*p<0.002 and **p<0.01). e-Survival rate curve of mice was followed up until 45 days after infection. Kaplan–Meier curves for survival of the different groups were compared using the log-rank test (all groups p<0,0001; groups ASP2+*T*.*cruzi* and ASP2 +αCXCR3+*T*.*cruzi* p = 0,0091). Results are shown as individual values and the mean ± SEM for each group (n = 4). One of two independent experiments is presented.

Since we have previously shown that the recirculation of CD8^+^ T lymphocytes was more important than their proliferative response to control *T*. *cruzi* infection [13], we evaluated which chemokine receptors could be important to drive the migration and to the effector functions of ASP2-specific CD8^+^ T-cells after vaccination of highly susceptible A/Sn mouse lineage [9] challenged with the virulent *T*. *cruzi* Y strain. In order to analyze that, immunized and infected mice were treated with chemokine-specific blocking monoclonal antibodies to the Th1-related chemokines CXCR3 and CCL2. On the same day of challenge with the Y strain infection, A/Sn mice were treated with the anti-CXCR3 or anti-CCL2 monoclonal antibodies. This procedure was repeated every 48 hours until day 20 after infection. We observed that the treatment with anti-CCL2 antibody had no impact on the protective effect of ASP2 vaccination, as parasitemia levels remained lower compared to the ASP2+*T*.*cruzi* group, whereas, as expected, higher parasitemia levels were observed in mice of group βgal+*T*.*cruzi* (Fig 2A). Further, all mice from untreated and anti-CCL2-injected ASP2+*T*.*cruzi* groups survived, while mice from βgal+*T*.*cruzi* group succumbed to infection (Fig 2B). To approach the participation of CCR2, which has as ligands CCL2 and other CC-chemokines [35], we used CCR2-deficient (*ccr2*^-/-^) mice. As previously described [36], *T*. *cruzi*-infected CCR2-deficient mice died due to infection, while wild-type resistant C57BL/6 mice survived. However, all CCR2-deficient mice immunized with the DNA/adenovirus ASP2 vaccine survived after to be challenged with *T*. *cruzi* (S2A and S2B Fig). After anti-CXCR3 administration into ASP2-vaccinated and challenged mice, we observed a trend in parasitemia increase only on day 12 after infection, when the peak of parasitemia was noticed, when compared to the immunized and isotype-treated control group (ASP2+*T*.*cruzi*), as shown in Fig 2C. At 20 days after infection, the quantification of parasite load in spleen by real time qPCR supported that trend in the treated group, showing that the number of parasites in the spleen of anti-CXCR3-treated vaccinated and challenged mice (ASP2+αCXCR3+*T*.*cruzi* group) was similar to the βgal+*T*.*cruzi* group and it contrasted with the low parasite load found in the spleen of mice of the isotype-treated ASP2+*T*. *cruzi* group (Fig 2D). The survival rate was followed for a 45-day period after infection and all mice from the ASP2+αCXCR3+*T*.*cruzi* group died due to infection while 100% of mice of the ASP2+*T*.*cruzi* group survived (Fig 2E). Taken together, these data indicate that the CXC-chemokine CXCR3 is important to control parasite dissemination and mice survival after challenge of ASP2-vaccinated mice.

### Anti-CXCR3 treatment did not alter the number of cytokine-producing specific CD8^+^ T cell in spleen

**Fig 3:**
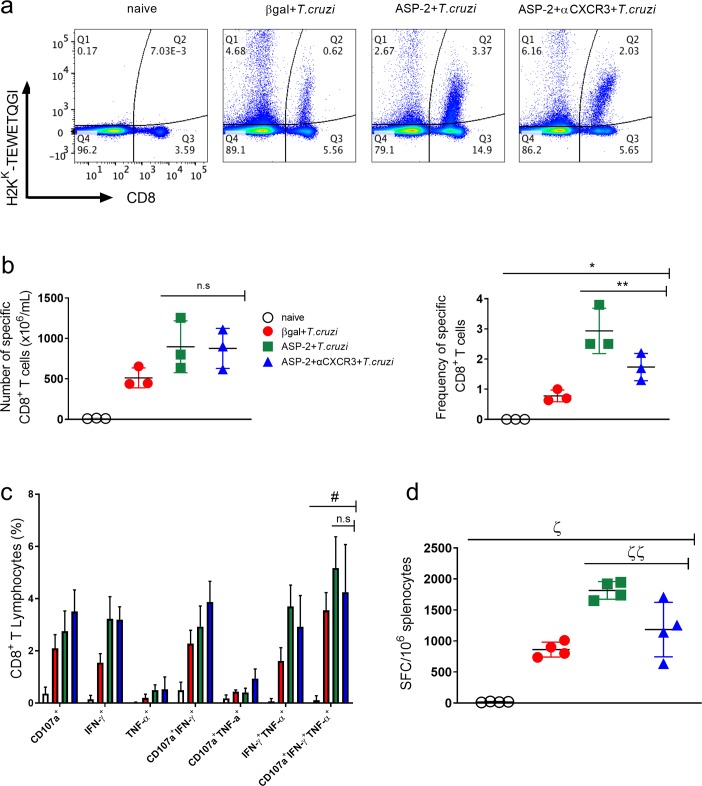
Anti-CXCR3 treatment did not alter the cytokine production by specific CD8^+^ T cells. Specific CD8^+^ T cells from the spleen were labeled using the dextramer H2K^K^-TEWETGQI and anti-CD8^+^ antibody. To measure cytokine production, splenocytes from βgal+*T*.*cruzi*, ASP2+*T*.*cruzi*, and ASP2+αCXCR3+*T*.*cruzi* were stimulated for 12 hours with TEWETGQI peptide of *T*. *cruzi*. a-Dot-plots show the frequency of specific H2K^K^-TEWETGQI CD8^+^ T cells in the spleen from each group. b-The frequency and absolute number of specific CD8^+^ T cells in the spleen of βgal+*T*.*cruzi*, ASP2+*T*.*cruzi*, and ASP2+αCXCR3+*T*.*cruzi* groups were quantified. c-The bar graph represents the percentage of CD8^+^ T cells expressing each individual molecule or the combinations after *ex vivo* stimulation (CD107a, IFN-γ and/or TNF-α). Boolean analysis was performed using FlowJo software, version 8.4. d-ELISpot graph with the number of IFN-γ producing cells. SFC = Spot-Forming Cell. Results are representative of two independent experiments with the mean ± SD of each individual group (n = 3). Statistical analysis was performed using the One-Way ANOVA and Tukey’s HSD tests. Symbols indicate that the values observed were significantly different between the groups (*p = 0,0002; **p<0.05; ^#^p = 0,001; ^ζ^p<0,0001; ^ζζ^p<0,05) and n.s means no significant.

Next, we analyzed the number of antigen-specific CD8^+^ T-cells after the treatment with anti-CXCR3 antibody. To perform that, we measured the number of specific CD8^+^ T-cells in spleen using the H-2K^k^-restricted TEWETGQI multimer, characterized as an immunodominant epitope of the ASP2 protein in A/Sn mice [3]. As expected, after immunization and infection, the frequency of TEWETGQI-specific CD8^+^ T-cells was higher in mice of ASP2+*T*.*cruzi* group than in mice of βgal+*T*.*cruzi* control group. After treatment with anti-CXCR3, we observed a decrease in the frequency of TEWETGQI-specific CD8^+^ T-cells in the anti-CXCR3 treated group, but no statistical differences in absolute numbers of TEWETGQI-specific CD8^+^ T-cells were observed in ASP2+αCXCR3+*T*.*cruzi* group when compared to the ASP2+*T*.*cruzi* group (Fig 3A and 3B), suggesting that the treatment with anti-CXCR3 antibody did not influence in the number of TEWETGQI-specific CD8^+^ T-cells in the A/Sn mice’s spleen. To investigate whether anti-CXCR3 treatment affected the polyfunctionality and cytokines production by TEWETGQI-specific CD8^+^ T-cells, we performed an Intracellular Staining assay (ICS) to measure the percentage of epitope-specific CD8^+^ T-cells producing IFN-γ and TNF cytokines as well as the degranulation marker CD107a molecule (LAMP-1), an indirect indicator of cytotoxicity activity, after *ex vivo* stimulation with TEWETGQI peptide. The gate strategy used to evaluate the polyfunctionality of TEWETGQI-specific CD8^+^ T-cells is in S3A and S3B Fig. After immunization and infection (ASP2+*T*. *cruzi*), the number of splenic polyfunctional (IFN-γ^+^TNF^+^CD107a^+^) TEWETGQI-specific CD8^+^ T-cells increased (5.17 ± 0.68), when compared to βgal+*T*.*cruzi* control group (3.55 ± 1.19) (Fig 3C). Also, we observed that the treatment with anti-CXCR3 did not alter the frequency of polyfunctional TEWETGQI-specific CD8^+^ T-cells (4.25 ± 1.82) in comparison to the isotype-treated ASP2+*T*.*cruzi* group (Fig 3C). Using the ELISpot assay to detect IFN-γ-secreting cells, we observed that the number of IFN-γ-producing CD8^+^ T-cells in βgal+*T*.*cruzi* group was lower than in the immunized and infected groups (Fig 3D). In addition, the number of IFN-γ-producing CD8^+^ T-cells decreased in ASP2+αCXCR3+*T*.*cruzi* when compared to ASP2+*T*.*cruzi* group. Collectively, these data provide evidence that the polyfunctionality capacity of TEWETGQI-specific CD8^+^ T-cells, characterized by the capacity of producing cytokines and degranulation at the same time, was not altered after anti-CXCR3 administration.

### Increased proapoptotic phenotype on TEWETGQI-specific CD8^+^ T-cells after anti-CXCR3 treatment

**Fig 4:**
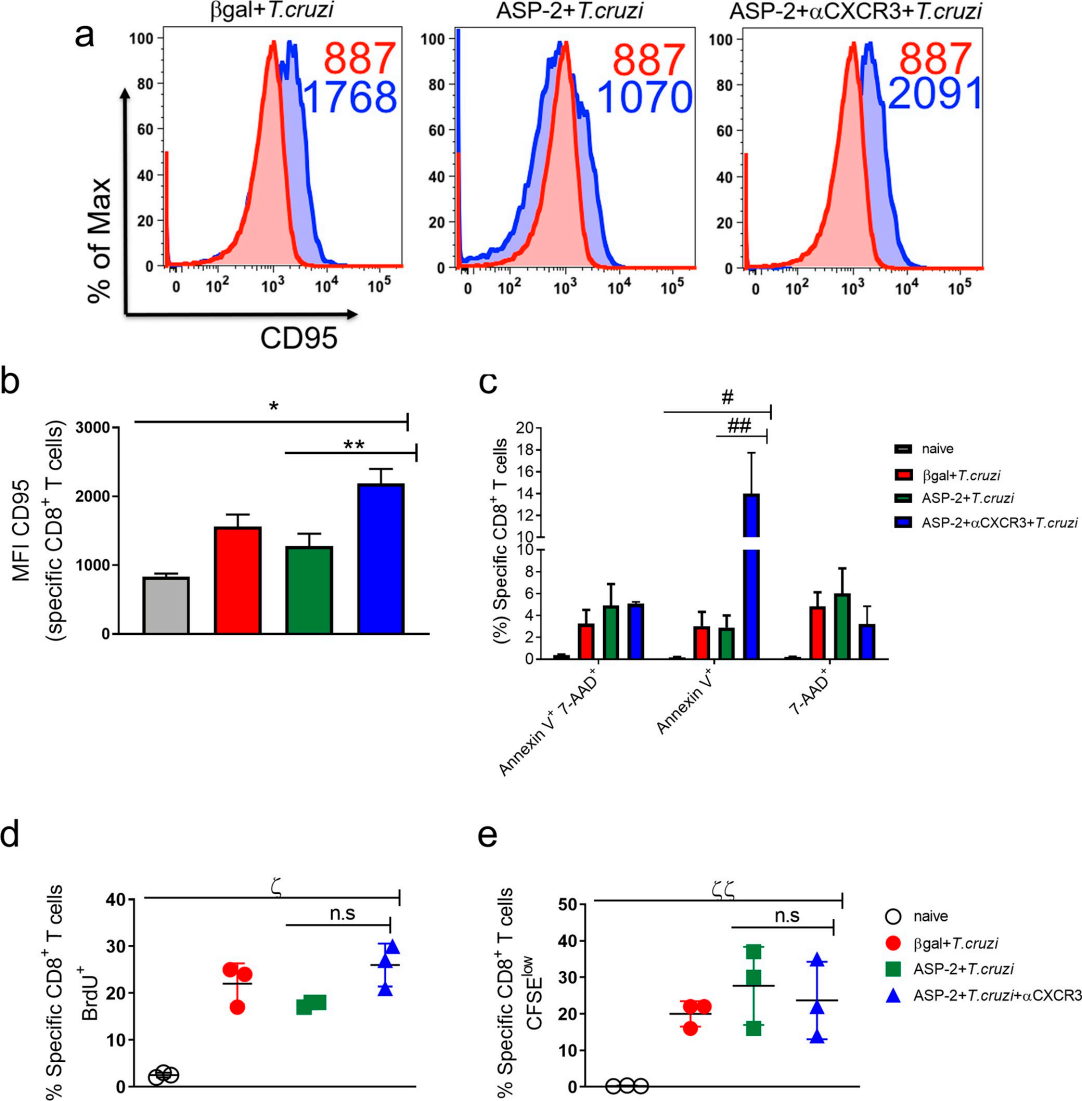
Anti-CXCR3 treatment increased the proapoptotic phenotype of specific CD8^+^ T cells, but those cells could proliferate. Specific CD8^+^ T cells from βgal+*T*.*cruzi*, ASP2+*T*.*cruzi*, and ASP2+ αCXCR3+*T*.*cruzi* groups were labeled to check proapoptotic profile. a-Histograms represent the MFI of CD95 (Fas) molecule on specific CD8^+^ T cells surface (from spleen). The red histogram represents the naïve group and the blue are: βgal+*T*.*cruzi*, ASP2+*T*.*cruzi* and ASP2+αCXCR3+*T*.*cruzi* groups. b-Bar graph shows the average of CD95 MFI on specific CD8^+^ T cells. c- Boolean analysis with the frequency of specific CD8^+^ T cells positive for annexin V^+^ and/or 7-AAD^+^. d-The percentage of specific CD8^+^ T cells that incorporated the BrdU molecule. The BrdU administration was done in the same day after infection (2mg of BrdU, via i.p route) and every 48 hours until 20 days after infection. e-Percentage of specific CD8^+^ T cells expressing CFSE^low^. The splenocytes from βgal+*T*.*cruzi*, ASP2+*T*.*cruzi* and ASP2+αCXCR3+*T*.*cruzi* groups were labeled with CFSE and stimulated for 5 days with TEWETGQI peptide. Afterword the specific CD8^+^ T cells were labeled with H2K^k^-TEWETGQI dextramer. Results are shown as individual values and as the mean ± SEM for each group (n = 3). Statistical analysis was performed using the One-Way ANOVA and Tukey’s HSD tests. Symbols indicate that the values observed were significantly different between the groups (*p < .0001; **p < .01; ^#^p < .0001; ^##^p<0.01; ^ζ^p < .0001 and ^ζζ^p = 0.0105) and n.s means no significant.

Previously, we have described that TEWETGQI-specific CD8^+^ T-cells generated by prime-boost heterologous immunization are effectors (TE), characterized by the CD44^high^, CD11a^high^, CD62L^low^, CD127^low^, and KLRG-1^high^ phenotype [12]. These cells play a crucial role in the control of infection by producing cytokines and killing the target cells by direct cytotoxicity [9]. Here, we evaluated whether anti-CXCR3 treatment affects the function-linked phenotypes of TEWETGQI-specific CD8^+^ T-cells in the spleen. In order to analyze that, TEWETGQI-specific CD8^+^ T-cells were labeled with tetramer and markers associated with cell activation and differentiation. Overall, we observed that anti-CXCR3 treatment did not alter the phenotype of TEWETGQI-specific CD8^+^ T-cells when compared to the isotype-treated ASP2+*T*.*cruzi* group. Indeed, TEWETGQI-specific CD8^+^ T-cells from the ASP2+αCXCR3+*T*.*cruzi* group had effector phenotype characterized as CD44^high^, CD11a^high^, CD62L^low^ and KLRG-1^high^, comparable to the epitope-specific CD8^+^ T-cells found in the ASP2+*T*.*cruzi* group (S4 Fig). Interestingly, we observed that the expression of the CD95 molecule was increased in the specific CD8^+^ T cells from spleen of ASP2+αCXCR3+*T*.*cruzi* group, when compared to the βgal+*T*.*cruzi* and ASP2+*T*.*cruzi* groups (Fig 4A and 4B). Previously, our group showed that the reason of a suboptimal CD8^+^ T-cell response profile during infection with *T*. *cruzi* was associated with an upregulation of CD95 expression and a proapoptotic phenotype, that was reversible with ASP2 vaccination which prevented that phenotyping observed only during infection [37]. Taking into account those findings, we evaluated the proapoptotic phenotyping by labeling specific CD8^+^ T cells with annexin V and 7-AAD molecules. We observed in ASP2+αCXCR3+*T*.*cruzi* group an increase in annexin V levels when compared to the βgal+*T*.*cruzi* and ASP2+*T*.*cruzi* groups, however, the percentage of cells expressing 7-AAD was similar in all groups (Fig 4C), indicating that anti-CXCR3 treatment increased the apoptotic phenotype in specific CD8^+^ T cells, but not necrosis. Next, we assessed the proliferative response of the TEWETGQI-specific CD8^+^ T-cells *in vivo* by BrdU incorporation assay and by CFSE-labeling after *ex-vivo* re-stimulation with TEWETGQI peptide. The number of epitope-specific CD8^+^ T-cells that incorporated BrdU was similar in all infected groups (Fig 4D). Similar results were also observed in *ex vivo* CFSE-labeled cell proliferation assay (Fig 4E). Together, these results suggest that anti-CXCR3 treatment of vaccinated and challenged mice increased the proapoptotic phenotype of TEWETGQI-specific CD8^+^ T-cells in the spleen.

### CXCR3 is important to cytotoxicity of specific CD8^+^ T cells

**Fig 5:**
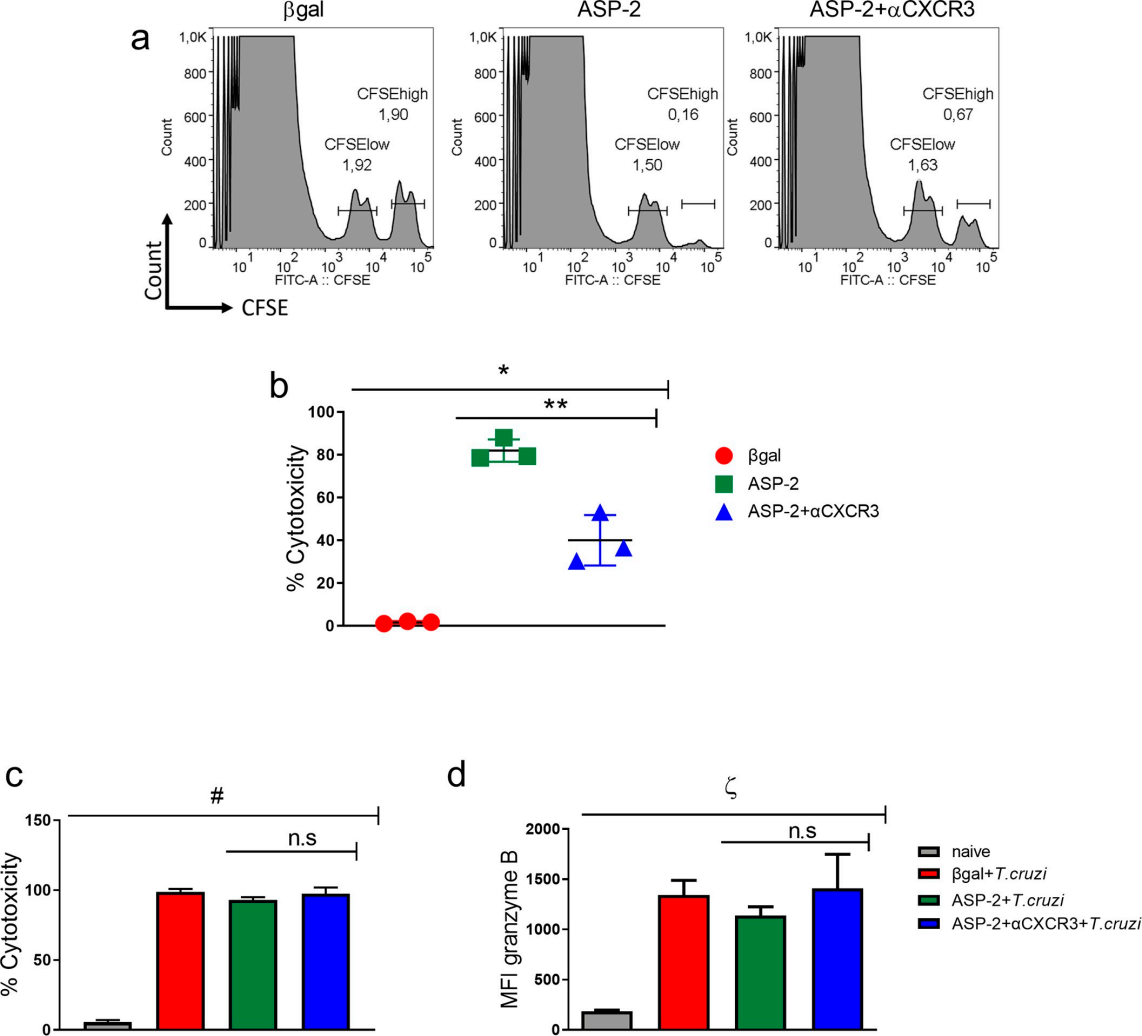
CXCR3 receptor is important to cytotoxicity of specific CD8^+^ T cells during the immunization. a-Histograms represent the frequency of CFSE^low^ and CFSE^high^ population that were transferred to βgal, ASP2, and ASP2+αCXCR3 groups. After 12 hours of transference, the percentage of CFSE^high^ cell lyses was calculated as described in the methods section. b-Cytotoxicity percentage of specific CD8^+^ T cells during immunization and treatment with anti-CXCR3. c-Percentage of cytotoxicity of specific CD8^+^ T cells during immunization, infection and treatment with anti-CXCR3 d-Granzyme B MFI on specific CD8^+^ T cells from βgal+*T*.*cruzi*, ASP2+*T*.*cruzi*, and ASP2+αCXCR3+*T*.*cruzi* groups. Results are shown as individual values and as the mean ± SEM for each group (n = 3). One of three independent experiments is presented. Statistical analysis was performed using the One-Way ANOVA and Tukey’s HSD tests. Symbols indicate that the values observed were significantly different between the groups (*p < .0001; **p < .01; ^#^p < .0001; ^ζ^p = 0.0001) and n.s means no significant.

One of the effector functions of the CD8^+^ T-cells is to kill infected cells by direct cytotoxicity, which is crucial for controlling infection by *T*. *cruzi* [9]. Here, we evaluated the cytotoxicity activity of TEWETGQI-specific CD8^+^ T-cells after treatment with anti-CXCR3 antibody after immunization and infection. The cytotoxicity assay was performed using transference of 1x10^7^ CFSE^low^ (not pulsed) and CFSE^high^ (pulsed with TEWETGQI peptide) to experimental groups. After 12 hours, the percentage of CFSE^high^ lysis was measured. We demonstrated that the percentage of cytotoxicity in immunized mice treated with the anti-CXCR3 blocking antibody (ASP2+αCXCR3) decreased when compared to isotype-treated ASP2-immunized mice (Fig 5A and 5B). After infection, however, no differences were observed in the cytotoxicity activity of CD8^+^ T-cells in the spleen of mice from βgal+*T*.*cruzi*, ASP2+*T*.*cruzi*, and ASP2+αCXCR3+*T*.*cruzi* experimental groups (Fig 5C). Moreover, granzyme B production by TEWETGQI-specific CD8^+^ T-cells was similar in these three experimental groups (Fig 5D). Overall, the CXC-chemokine receptor CXCR3 indicates to be important to the cytotoxicity activity of TEWETGQI-specific CD8^+^ T-cells generated after prime-boost immunization protocol in A/Sn mice.

### CXCR3 ligands are selectively expressed in the heart tissue of vaccinated and challenged mice

**Fig 6:**
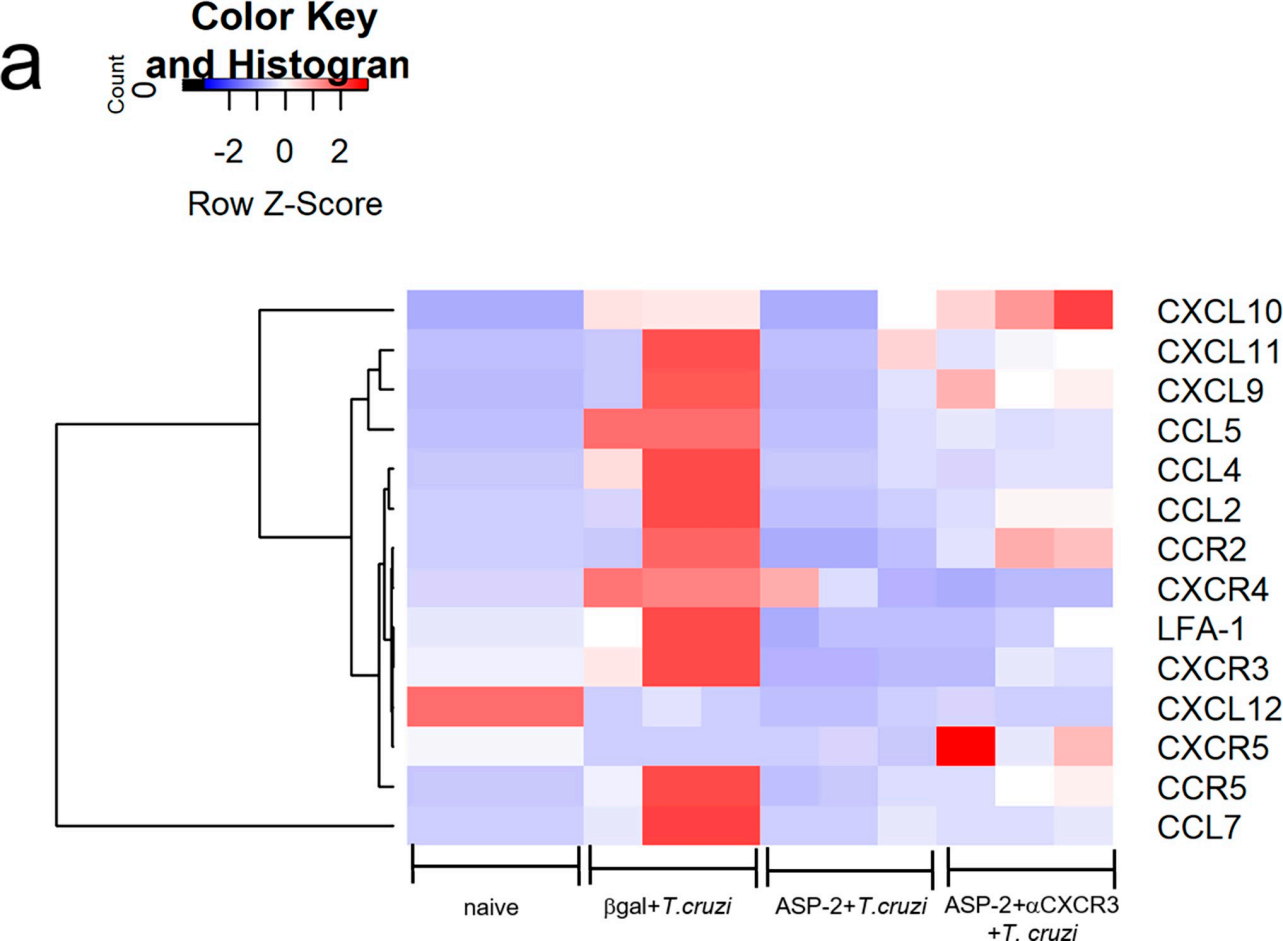
CXCR3 ligands were expressed in heart of infected mice. a-The heatmap graph represents the fold change of chemokine genes in heart of βgal+*T*.*cruzi*, ASP2+*T*.*cruzi*, and ASP2+αCXCR3+*T*.*cruzi* groups. The quantification of chemokines gene expression in heart was on day 20 after infection and we used a customized plate (Mouse Chemokines Plate targets genes). Results are shown as individual values and the mean ± SEM for each group (n = 3).

Having shown the high expression of CXCR3 chemokine receptor in TEWETGQI-specific CD8^+^ T-cells, we evaluated the expression of CXCR3 ligands in heart, as well as other molecules involved in cell migration (CC-chemokines and their receptors and cell adhesion molecules). In heart of naïve mice, all genes had low expression, except CXCL12 gene that was downregulated in infected groups. In general, we observed that in infected heart of mice from βgal+*T*.*cruzi* group only CXCL12 and CXCR5 were low expressed, while the other genes were highly expressed, including CXCR3 ligands such as: CXCL11, CXCL10 and CXCL9. In ASP2+*T*.*cruzi* experimental group, we observed a low expression of inflammatory cell migration genes, whereas in heart of mice from ASP2+αCXCR3+*T*.*cruzi* group, CXCR3 ligands CXCL10 and CXCL9 were high expressed, as well as CCR2 and CXCR5 (Fig 6A). These results suggest that CXCR3 ligands, CXCL10 and CXCL9 were selectively high expressed in heart of infected mice, supporting that vaccination prevented the expression of most of the genes involved in cell migration here studied.

### CXCR3 guides specific CD8^+^ T cells into infected heart tissue

**Fig 7:**
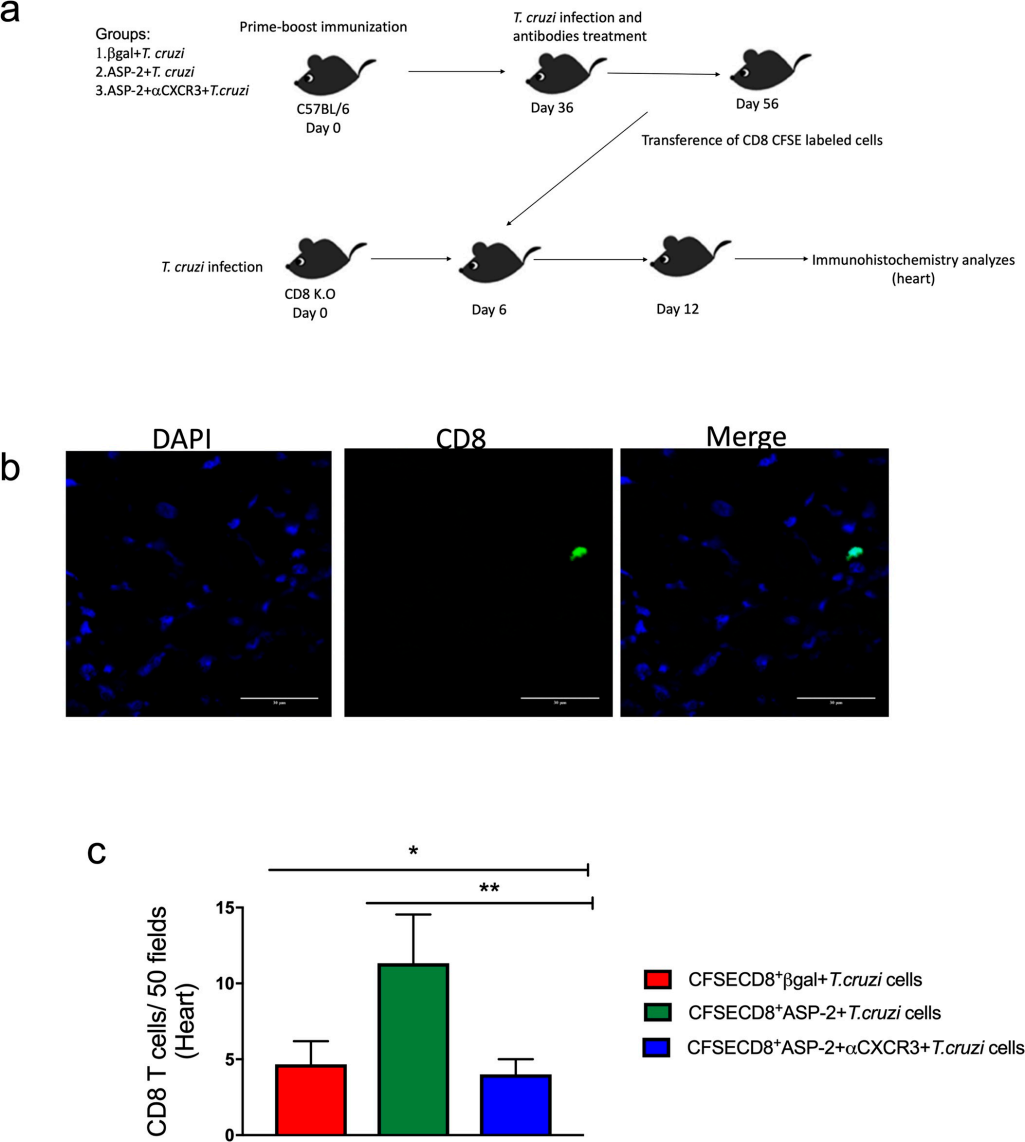
The treatment with anti-CXCR3 decreases the number of CD8^+^ T cells in the heart. a-Experimental design showing the immunization, infection, treatment with anti-CXCR3 antibody and adoptive transference of CD8^+^ CFSE labeled cells to CD8 deficient mice (infected). Briefly, C57BL/6 mice were immunized with prime-boost heterologous protocol, the first dose of immunization was with pCDNA3/pIgSPclone9 after 21 days of prime the mice received Adβgal/AdSP-2 and after 15 days were infected with *T*. *cruzi* and treated with 250 μg of CXCR3 antibody, and on day 20 after infection, CD8^+^ T cells from spleen were purified, labeled with CFSE, and transferred into CD8 K.O mice previously infected (on day 6 after infection). After 6 days of transference, the number of CD8^+^ T cells in heart from βgal+*T*.*cruzi*, ASP2+*T*.*cruzi* and ASP2+αCXCR3+*T*.*cruzi* groups was quantified. b-IHC in heart showing the CFSE^+^CD8^+^ T cells (green). The DAPI staining was used to label the nucleus of the cells. c-Number CFSE^+^CD8^+^ T cells that migrated to CD8 K.O mice hearts. The cells were counted using the fluorescent microscopy and 50 fields were counted. Results are shown as individual values and the mean ± SEM for each group (n = 3). Statistical analysis was performed using the One-Way ANOVA and Tukey’s HSD tests. Symbols indicate that the values observed were significantly different between the groups (*p = 0.010; **p < .05).

*T*. *cruzi* infects the cardiac tissue [38] triggering an inflammatory response associated with tissue injury, leading to cardiomyopathy in 30% of the infected patients in the chronic phase of Chagas disease [11,39]. Thus, we evaluated the migration of CD8^+^ T-cells to heart after anti-CXCR3 treatment. For that propose, CFSE-labeled CD8^+^ T-cells obtained from βgal+*T*.*cruzi*, ASP2+*T*.*cruzi*, or ASP2+αCXCR3+*T*.*cruzi* groups were transferred to groups of CD8-deficient mice (*cd8*^-/-^) as shown in the experimental scheme in Fig 7A. The number of CFSE^+^CD8^+^ T-cells was quantified, and we observed a statistical decreased in the number of CFSE^+^CD8^+^ T-cells that migrated in the heart tissue of CD8-deficient mice who received CD8^+^ T-cells from ASP2+αCXCR3+*T*. *cruzi* mice, when compared to the recipient mice that received cells from the ASP2+*T*.*cruzi* donors (Fig 7B and 7C).

**Fig 8:**
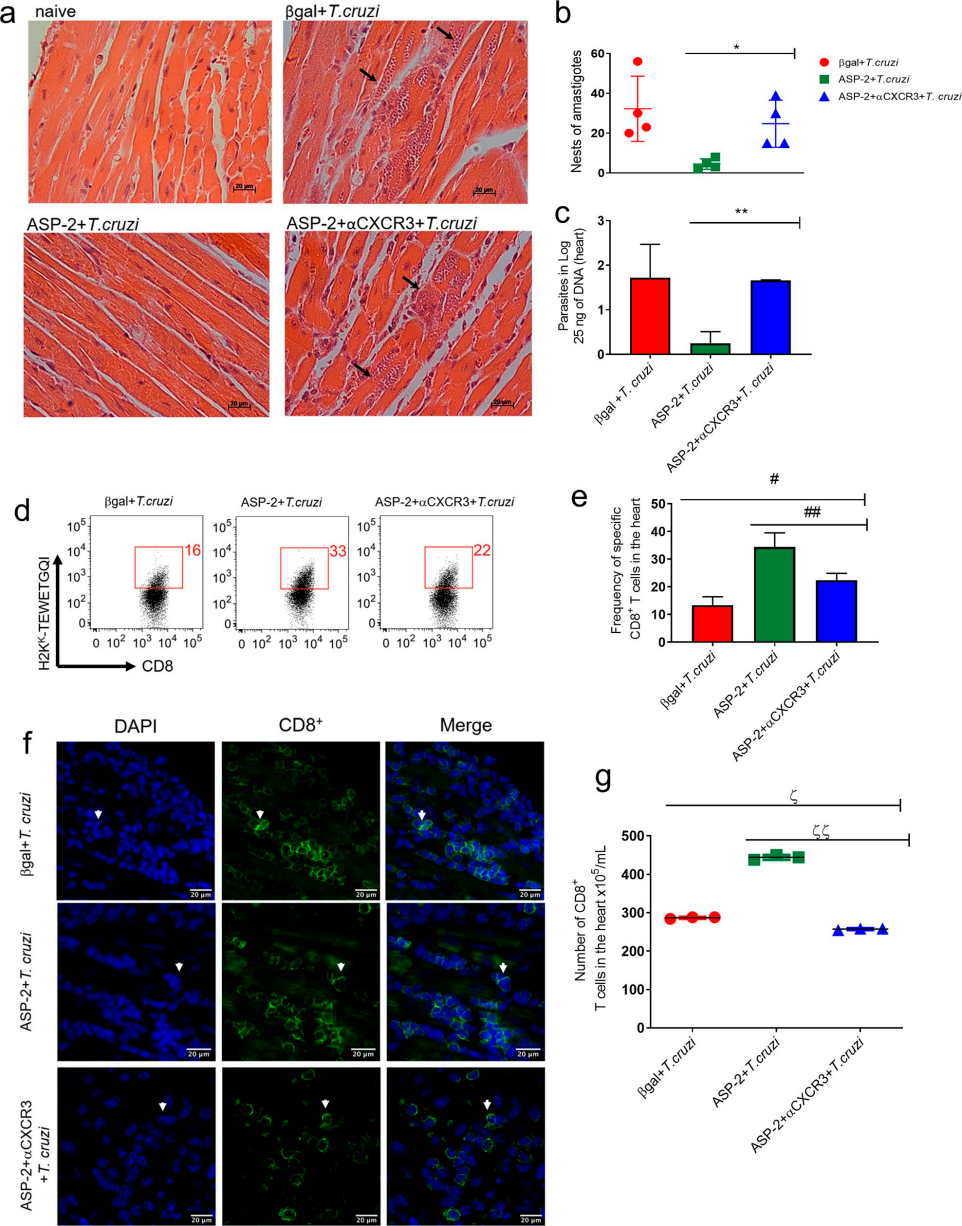
The migration of specific CD8^+^ T cells to heart was impaired after treatment with anti-CXCR3 antibody. a-Histological section from heart tissue: the black arrows show the amastigotes nests in βgal+*T*.*cruzi*, ASP2+*T*.*cruzi*, and ASP2+αCXCR3+*T*.*cruzi* groups. b-Graph represents the number of amastigotes nests in the heart, measured in 25 fields using light microscopy. c-Number of parasites in heart tissue quantified by Real time PCR from 25 ng of DNA sample. d-The dot-plot show the frequency of specific CD8^+^ T cells in the heart tissue. e-The frequency average of specific CD8^+^ T cells in heart. f-IHC section showing CD8^+^ T cells (in green) in cardiac tissue on day 20 after infection. The DAPI was used to reveal the CD8^+^ T cell nucleus. g-Number of CD8^+^ T cells in cardiac tissue. Results are shown as individual values and the mean ± SEM for each group (n = 3). Statistical analysis was performed using the t-test, One-Way ANOVA, and Tukey’s HSD tests. Symbols indicate that the values observed were significantly different between the groups (*p = 0.0251; **p < .0001; ^#^p = 0.001; ^##^p = 0.001; ^ζ^p < .0001 and ^ζζ^p < .01).

To endorse these results, we measured parasite burden and migration of TEWETGQI-specific CD8^+^ T-cells into the heart, after vaccination, challenge and anti-CXCR3 antibody administration. Firstly, we quantified the number of amastigote nests in the heart tissue by HE (hematoxylin and eosin) staining, and we observed that both βgal+*T*.*cruzi* and ASP2+αCXCR3+*T*.*cruzi* experimental groups had higher number of amastigote nests when compared to the ASP2+*T*.*cruzi* group (Fig 8A and 8B). Also, we estimated the parasite load using qPCR, and again both βgal+*T*.*cruzi* and ASP2+αCXCR3+*T*.*cruzi* groups had an increased number of parasites in heart tissue when compared to the ASP2+*T*.*cruzi* group (Fig 8C). Considering the results described above and the increased numbers of parasite nests seen in the heart after treatment with anti-CXCR3 antibody, we evaluated whether TEWETGQI-specific CD8^+^ T-cells migrate into the heart tissue after anti-CXCR3 treatment. For that propose, we purified parasite-specific CD8^+^ T-cells from cardiac tissue using a pool of dissociated hearts (n = 5 mice/group) and those cells were labeled with the H-2K^k^-restricted TEWETGQI multimer. Curiously, the frequency of TEWETGQI-specific CD8^+^ T-cells decreased in the heart tissue of ASP2+αCXCR3+*T*.*cruzi* group when compared to ASP2+*T*.*cruzi* group (Fig 8D and 8E), whereas the βgal+*T*.*cruzi* group had the lowest frequency of TEWETGQI-specific CD8^+^ T-cells. Additionally, we quantified the number of CD8^+^ T-cells in heart using confocal microscopy. Again, anti-CXCR3 decreased the number of CD8^+^ T cells in the heart (Fig 8F and 8G). Altogether, these results show that CXCR3 guides TEWETGQI-specific CD8^+^ T-cells toward the *T*. *cruzi*-infected heart tissue, and these cells play an important role controlling the infection.

## Discussion

The recirculation of T lymphocytes into infected and, frequently, injured sites is essential for controlling infection by *T*. *cruzi* [13]. As chemokine receptors are pivotal for T-cell migration, we hypothesized that CXCR3 receptor might play an important role in parasite-specific CD8^+^ T-cells migration into infected tissues after immunization and challenge by *T*. *cruzi*. Firstly, we evaluated CCR2 and CXCR3 role during immunization and infection and both CXCR3 and CCR2 receptors are highly expressed in the heart of *T*. *cruzi* infected mice [22]. Other studies using the Colombian strain of *T*. *cruzi* have shown that CCR2-deficiency leads to increase in parasitemia [36]. The CC-chemokine receptor CCR2 is responsible for monocytes migration during the inflammation [40], being CCL2 (MCP-1) its main ligand. However, in our experiments the treatment with anti-CCL2 did not impact in parasitemia or survival rate. As CCR2-deficient mice were high susceptible and CCR2 has other ligands than CCL2 [41], we immunized CCR2-deficient mice, and all vaccinated mice survived to the challenge with *T*. *cruzi* infection. Moreover, after anti-CXCR3 treatment, all mice had an increased parasitemia and burden of tissue parasitism, and died due to infection, showing that CXCR3, but not CCR2, had a pivotal role in *T*. *cruzi* resistance. The role of CXCR3 in the resistance against infections by virus and other pathogens has been shown [42,26], reinforcing that CXCR3 is essential to control infection by intracellular pathogens.

CXCR3 is highly expressed in murine Th-1 CD4^+^ and CD8^+^ T-cells [43], and the CXC-chemokine receptor CXCR3 plays a role in the regulation of leukocyte migration into inflammatory sites in mice and human [44]. Here, we have shown that CXC-chemokine receptor CXCR3 is more highly expressed on TEWETGQI-specific CD8^+^ cells of *T*. *cruzi* challenged mice than in ASP2 immunized animals; however, the expression on effector CD4^+^ T-cells was similar between the groups. In addition, we found increased levels of ligands CXCL9 and CXCL10 in the serum of those mice. Although specific CD8^+^ T cells infected expressed higher levels of CXCR3 on cell surface, CD8^+^ T-cells from the immunized group had a higher migration index compared to specific CD8^+^ T cells generated only by infection, after the *ex vivo* stimulation with CXCL9 and CXCL10, but not with CXCL11. These three ligands are all induced by IFN-γ [45], but are differently expressed [46] and that may explain the differential role played by the CXC-chemokine receptor CXCR3 in several diseases [47].

Concerning the *in vitro* high migration capacity of CD8^+^ T cells of the immunized group, CXCR3 low expression in those cells may be explained because specific CD8^+^CXCR3^+^ cells from the spleen migrated to the non-lymphoid peripheral tissue, for example, the heart tissue. We observed a higher number of specific CD8^+^ T cells in the immunized group compared to the infected group. Another explanation might be that CXCR3 receptor from the immunized group is more responsive to CXCR3 ligands and the receptor is activated and internalized [48], which decreases the number of cells positive for CXCR3 receptor.

Additionally, we evaluated the effector function of the TEWETGQI-specific CD8^+^ T-cells after anti-CXCR3 antibody administration. We observed that these parasite-specific CD8^+^ T-cells present in the spleen can release cytokines in the ELISpot assay, in which we observed a decrease in the number of IFN-γ producing cells. However, in ICS assay, the percentage of IFN-γ CD8^+^ producing cells was similar in the ASP2 immunized group, indicating that the decrease was due to a technique variation. In addition, TEWETGQI-specific CD8^+^ T-cells after anti-CXCR3 antibody administration showed proliferative response. Similar results were observed during infection by virus and during anti-CXCR3 treatment [42,49–50]. However, during autoimmune diseases and infection by *Leishmania major*, CXCR3 is important for cytokine production and proliferation by CD8^+^ T-cells [51–53]. In addition to cytokine production, we evaluated the cytotoxicity of these TEWETGQI-specific CD8^+^ T-cells and during infection these cells show high cytotoxicity [32]; therefore, we decided to perform the analyzes in parasite-specific CD8^+^ T-cells generated only by immunization because during the infection the cells are very cytotoxic and it is difficult observed differences among the groups [3]. Our results showed that the treatment with anti-CXCR3 also decreased cytotoxicity of TEWETGQI-specific CD8^+^ T-cells in ASP2 immunized mice, corroborating the results observed by Thapa and colleagues [54]. Unlike this study, however, we did not observe a decrease in granzyme B production by these TEWETGQI-specific CD8^+^ T-cells. The decreased cytotoxicity activity may be explained because the CXC chemokine receptor CXCR3 is important to the contact between infected target cells and specific CD8^+^ T-cells [50].

The role of CXCR3 in the differentiation of CD8^+^ T-cells in memory subtypes has been shown in other studies [28]. The receptor CXCR3 is important for the intranodal positioning of T-cells and Th cell polarization [55] and facilitates CD8^+^ T-cell differentiation into short-lived effector cells and memory generation [27]. The TEWETGQI-specific CD8^+^ T-cells generated by heterologous immunization and challenge by *T*. *cruzi* infection are effector cells characterized by the expression of these molecules and levels: CD11a^high^, CD62L^low^, CD44^high^ and CD127^low^ and KLRG1^high^ [12]. The treatment with anti-CXCR3 did not alter the effector phenotype of the TEWETGQI-specific CD8^+^ T-cells but increased the levels of CD95 expression on cell surface of those cells. Previously, our group showed that TEWETGQI-specific CD8^+^ T-cells generated by immunization had lower CD95 expression when compared to cells generated after infection [37]. As CD95 is a cell death-promoting molecule [56], we analyzed the apoptosis in TEWETGQI-specific CD8^+^ T-cells and we observed an increase in annexin V expression, suggesting that anti-CXCR3 treatment increased the proapoptotic phenotype of TEWETGQI-specific CD8^+^ T-cells. The protection of cell death during immunization with ASP2 ensures that TEWETGQI-specific CD8^+^ T-cells trigger the effector function and control parasites replication. We have also demonstrated that CXCR3 molecule expression protected TEWETGQI-specific CD8^+^ T-cells from cell death.

CXCR3 receptor is also essential for CD8^+^ T-cell migration after immunization and, particularly, for parasite-specific CD8^+^ T-cell migration into the heart tissue after immunization and challenge with *T*. *cruzi* infection. Indeed, after anti-CXCR3 treatment, we observed a reduced number of CD8^+^ T-cells infiltrating the heart tissue. Consistently with the reduced number of CD8^+^ T-cells in the heart tissue of anti-CXCR3-treated mice, we found a significant increase in the number of amastigote nests and parasite load in the heart tissue of these mice. Furthermore, we demonstrated that several chemokines genes in infected mice hearts were highly expressed, indicating a high inflammation/migration to heart tissue. The high expression of CCL5/RANTES, CCL3/MIP-1a, CCL4/MIP-1b, CCL2/MCP-1 and the CXC chemokines CXCL10/IP-10 and CXCL9/MIG mRNA also have been detected in the heart tissue of acutely and chronically *T*. *cruzi*-infected mice [29].

The high expression of chemokines genes in the infected group did not guarantee a high migration of specific CD8^+^ T cells. Studies have shown that a pro-inflammatory environment in the heart tissue is sufficient to activate autoreactive T cells and cause cardiomyopathy during Chagas disease [57]. Immunization with ASP2 prevents the expression of most of the analyzed chemokines; however, expression of CXCR3 ligands CXCL9 and CXCL10 was observed in two animals. Also, both chemokines were detected in the mice’s serum, suggesting that parasite-specific CD8^+^ T-cells expressing CXCR3 can be attracted to the heart tissue. In fact, the number of TEWETGQI-specific CD8^+^ T-cells in the ASP2 immunized heart tissue is higher than in the infected group. In addition, in the ASP2 immunized group, the number of amastigote nests and parasite burden is lower than in the infected mice group, suggesting that the higher number of TEWETGQI-specific CD8^+^ T cells participate in the infection control. After anti-CXCR3 administration, we observed a high expression of CXCR3 ligands, which may be explained for the competition between anti-CXCR3 and the ligands to CXCR3 ligation, resulting in CXCR3 ligands accumulation.

The importance of CXCR3 in T cells migration has been demonstrated in several studies and shown that anti-CXCR3 treatment is effective at preventing acute and chronic heart rejection after transplantation [58]. Although previous studies have shown CXCR3 role in the migration of effector lymphocytes involved in the control of viral, protozoan and bacterial infection [49–60], our study reveals for the first time that CXCR3 receptor is pivotal for the migration and positioning of pathogen-specific CD8^+^ T-cells directly involved in the clearance of *T*. *cruzi* after the prime-boost immunization and challenge. Therefore, our data support that prime-boost vaccination protocol was effective in the selective CXC-chemokine-mediated CXCR3-driven activation, migration and positioning in a target tissue that is drastically affected during the chronic phase of *T*. *cruzi* infection. Moreover, our work places CXCR3 as a powerful molecule able to address specific cell to target tissue of infection and, therefore, to be included as a key requirement for design of vaccines against intracellular pathogens. The potential use of chemokines receptor as an adjuvant in vaccines strategies has been demonstrated in the dengue model [61]. In Chagas disease, CXCR3 receptor may be used to guide specific CD8^+^ T cells to the heart and prevent cell death. Consequently, it might control parasites replication.

In general, we have demonstrated that anti-CXCR3 treatment increased the susceptibility of immunized A/Sn mice, which died very quickly due to infection. Moreover, specific CD8^+^ T-cells decreased the migration into the heart tissue, and those cells displayed a pro-apoptotic profile. Taken together, those results show that CXCR3 has a critical role in the protective immune response and understanding its migratory role might support the development of vaccines against intracellular parasites such as *Trypanosoma cruzi*.

## Supporting information

S1 Fig During infection and/or immunization with ASP2 gene the levels of MCP-1 and RANTES chemokines were low. a-Quantity of MCP-1 and (b) RANTES chemokines in pg/mL in serum of naïve, βgal+*T*.*cruzi* and ASP-2+*T*.*cruzi*. The chemokines were measured on days zero, 6, 8,10, 12 and 14 after infection by Luminex assay. Results are shown as individual values and the mean ± SEM for each group (n = 4). One independent experiment is presented.
(TIF)

S2 Fig Prime-boost immunization protected CCR2 deficient mice against infection with *T. cruzi*. C57BL/6 or CCR2 deficient mice were immunized with heterologous prime-boost protocol, and 15 days after the last dose of immunization, mice were challenged with 1x10^4^ blood forms of Y strain of *T*. *cruzi*. a-Parasitemia in log of C57BL/6 and CCR2 deficient mice infected or immunized as described in the figure. b-Survival rate curve of mice was followed up until 30 days of infection. Results are shown as individual values and the mean ± SEM for each group (n = 4). One of two independent experiments is presented. The n.s means no differences on parasitemia levels between C57BL/6 and CCR2 K.O infected mice were found.
(TIF)

S3 Fig Gates strategy used for intracellular staining of cytokines analysis. A/Sn mice were immunized with ASP2 using the heterologous prime-boost vaccination regimen, infected with 150 tripomastigotes forms of *T*. *cruzi* and treated with anti-CXCR3 until the day 20^th^ after infection. At this point, the splenic cells were re-stimulated *ex vivo* in the presence of peptide TEWETGQI at a final concentration of 10 μM. After 12h, cells were stained with anti-CD8, anti-IFN-γ, and anti-TNF-α antibodies. a-Gate strategy was made as follows: SSC-A/Time, SSC-A/FSC-A, FSC-H/FSC-A and SSC-A/CD8. b-Dot-plot graphs represent the gate strategy used to analyze the production of intracellular cytokines in peptide-stimulated CD8^+^ T cells.
(TIF)

S4 Fig Treatment with anti-CXCR3 monoclonal antibody did not alter the effector phenotype of specific CD8+ T cells. Specific CD8^**+**^ T cells were labeled in the spleen using the dextramer H2K^K^-TEWETGQI with APC-fluorophore and all markers showed in the histograms. a-Histograms represent one animal of each group (βgal+*T*.*cruzi*, ASP-2+*T*.*cruzi*, and ASP-2+αCXCR3+*T*.*cruzi*). The MFI of each marker expressed on the surface of specific CD8^+^ T cells in the spleen were shown in the histograms. The markers that we choose are related with the activation and stimulation of T lymphocytes. Previously, our group described the effector phenotype on specific CD8^+^ T cells, as CD44^high^, CD62L^low^ and CD11a^high^. The red histogram represents the naïve group and the blue the groups βgal+*T*.*cruzi*, ASP-2+*T*.*cruzi*, and ASP-2+αCXCR3+*T*.*cruzi*. Results are shown as individual values and as the mean ± SEM for each group (n = 4).
(TIF)
