# Supplementary figures and images for: CXCR3 chemokine receptor guides Trypanosoma cruzi-specific T-cells triggered by DNA/adenovirus ASP2 vaccine to heart tissue after challenge
Source: PLoS Negl Trop Dis. 2019 Jul 29;13(7):e0007597. doi: 10.1371/journal.pntd.0007597 (PMC6687206; doi:10.1371/journal.pntd.0007597)

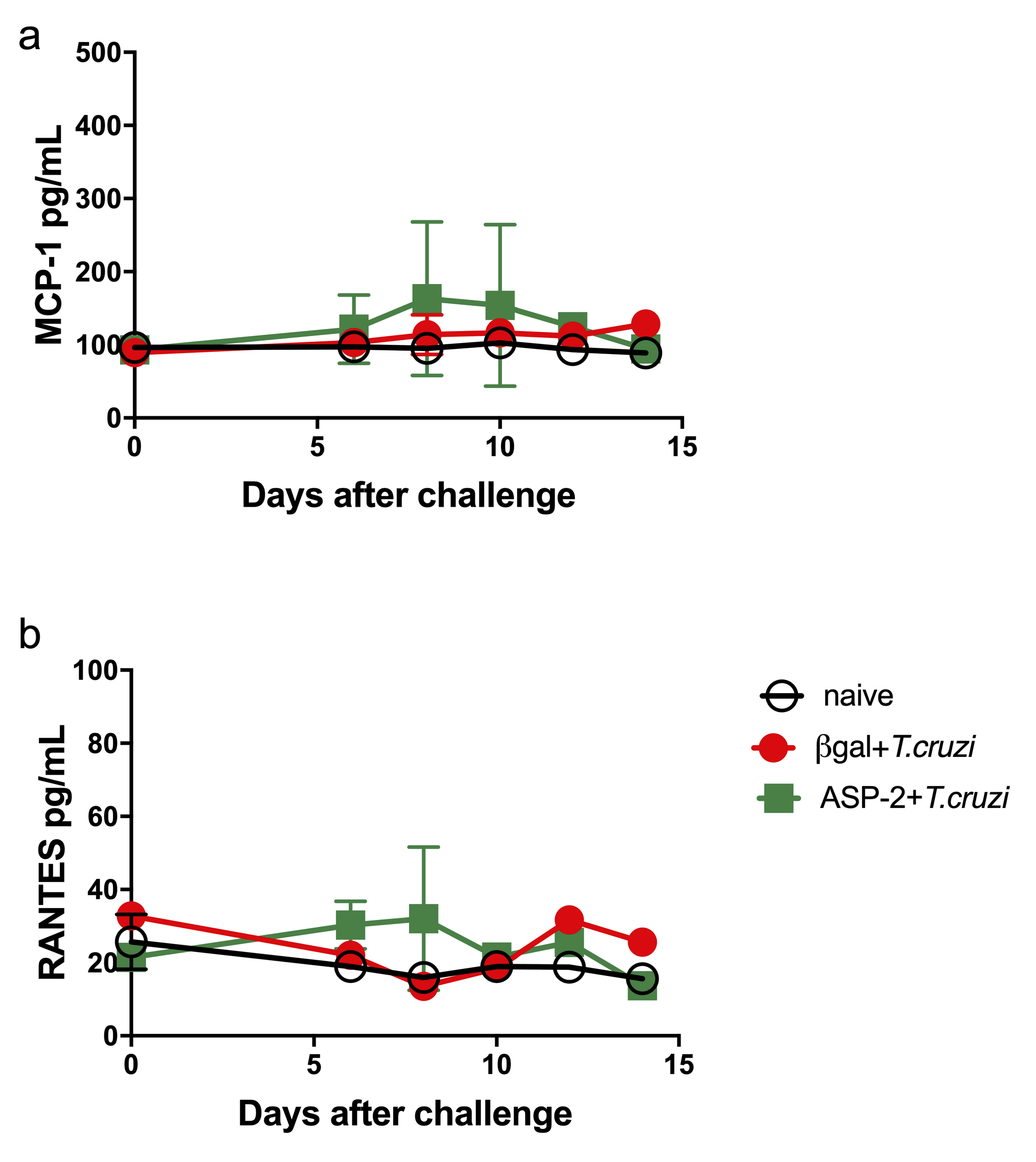

Supplement: S1 Fig — a-Quantity of MCP-1 and (b) RANTES chemokines in pg/mL in serum of naïve, βgal+T.cruzi and ASP-2+T.cruzi. The chemokines were measured on days zero, 6, 8,10, 12 and 14 after infection by Luminex assay. Results are shown as individual values and the mean ± SEM for each group (n = 4). One independent experiment is presented. (TIF) [file pntd.0007597.s001.tif]

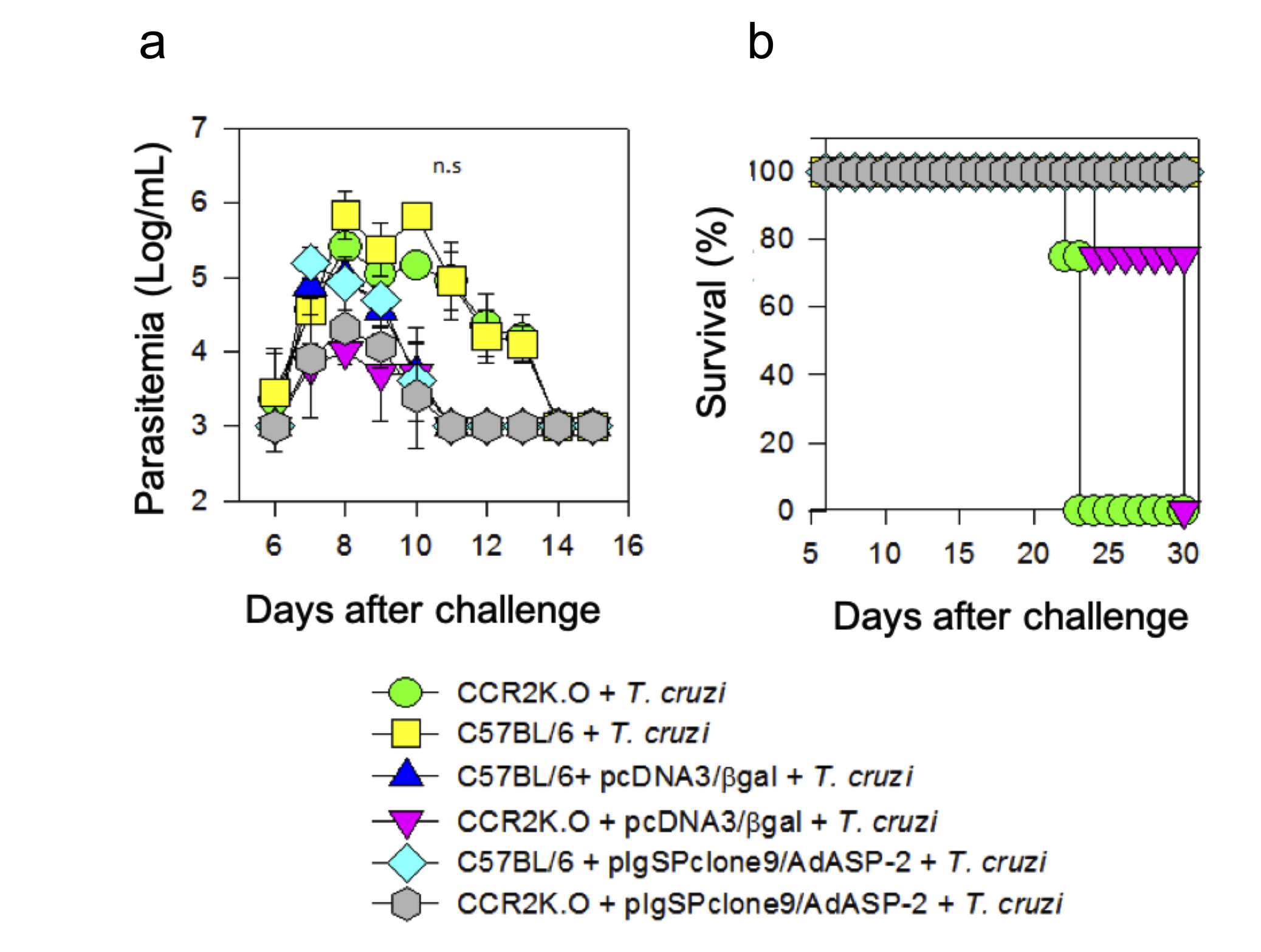

Supplement: S2 Fig — C57BL/6 or CCR2 deficient mice were immunized with heterologous prime-boost protocol, and 15 days after the last dose of immunization, mice were challenged with 1x10^4^ blood forms of Y strain of T. cruzi. a-Parasitemia in log of C57BL/6 and CCR2 deficient mice infected or immunized as described in the figure. b-Survival rate curve of mice was followed up until 30 days of infection. Results are shown as individual values and the mean ± SEM for each group (n = 4). One of two independent experiments is presented. The n.s means no differences on parasitemia levels between C57BL/6 and CCR2 K.O infected mice were found. (TIF) [file pntd.0007597.s002.tif]

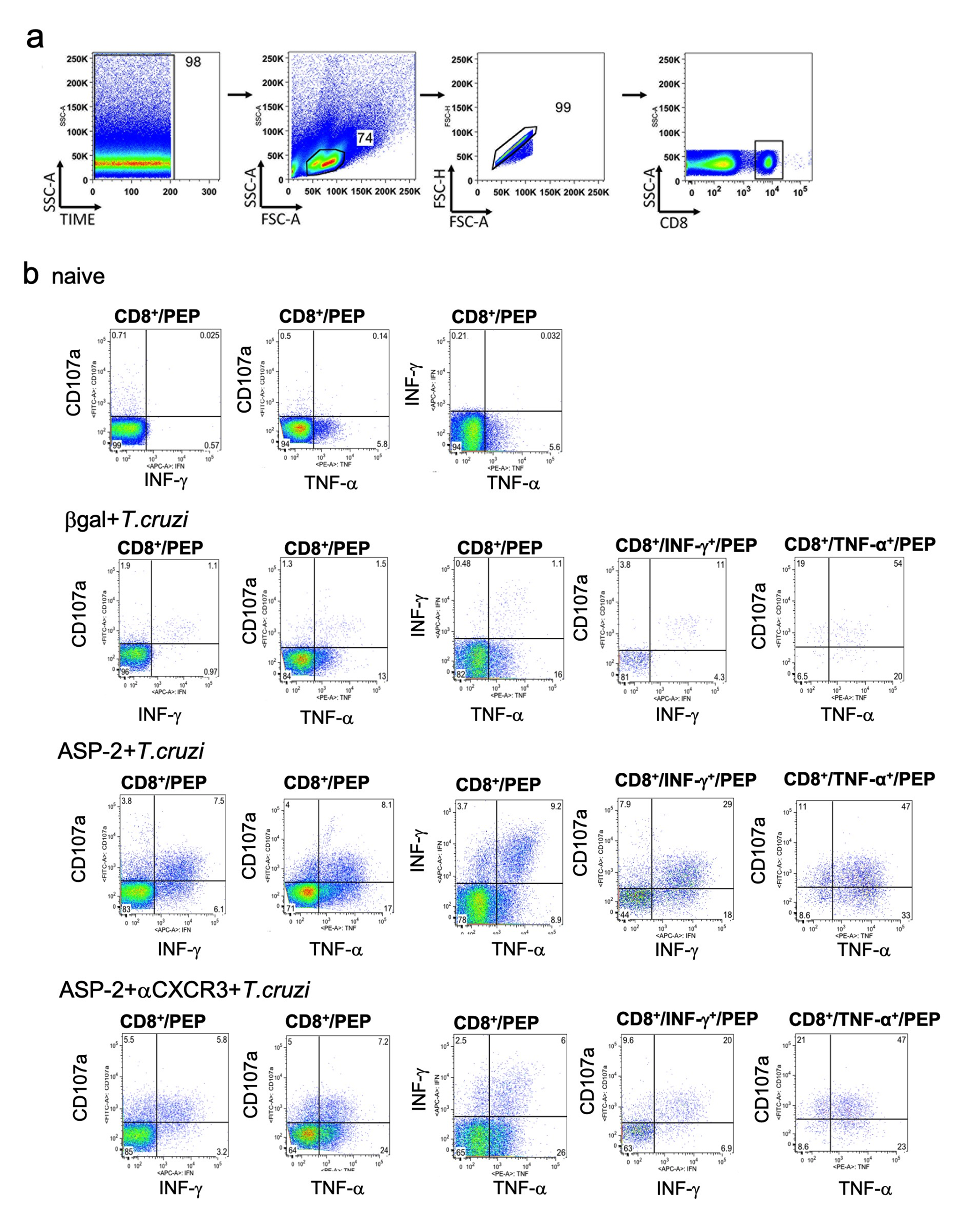

Supplement: S3 Fig — A/Sn mice were immunized with ASP2 using the heterologous prime-boost vaccination regimen, infected with 150 tripomastigotes forms of T. cruzi and treated with anti-CXCR3 until the day 20th after infection. At this point, the splenic cells were re-stimulated ex vivo in the presence of peptide TEWETGQI at a final concentration of 10 μM. After 12h, cells were stained with anti-CD8, anti-IFN-γ, and anti-TNF-α antibodies. a-Gate strategy was made as follows: SSC-A/Time, SSC-A/FSC-A, FSC-H/FSC-A and SSC-A/CD8. b-Dot-plot graphs represent the gate strategy used to analyze the production of intracellular cytokines in peptide-stimulated CD8+ T cells. (TIF) [file pntd.0007597.s003.tif]

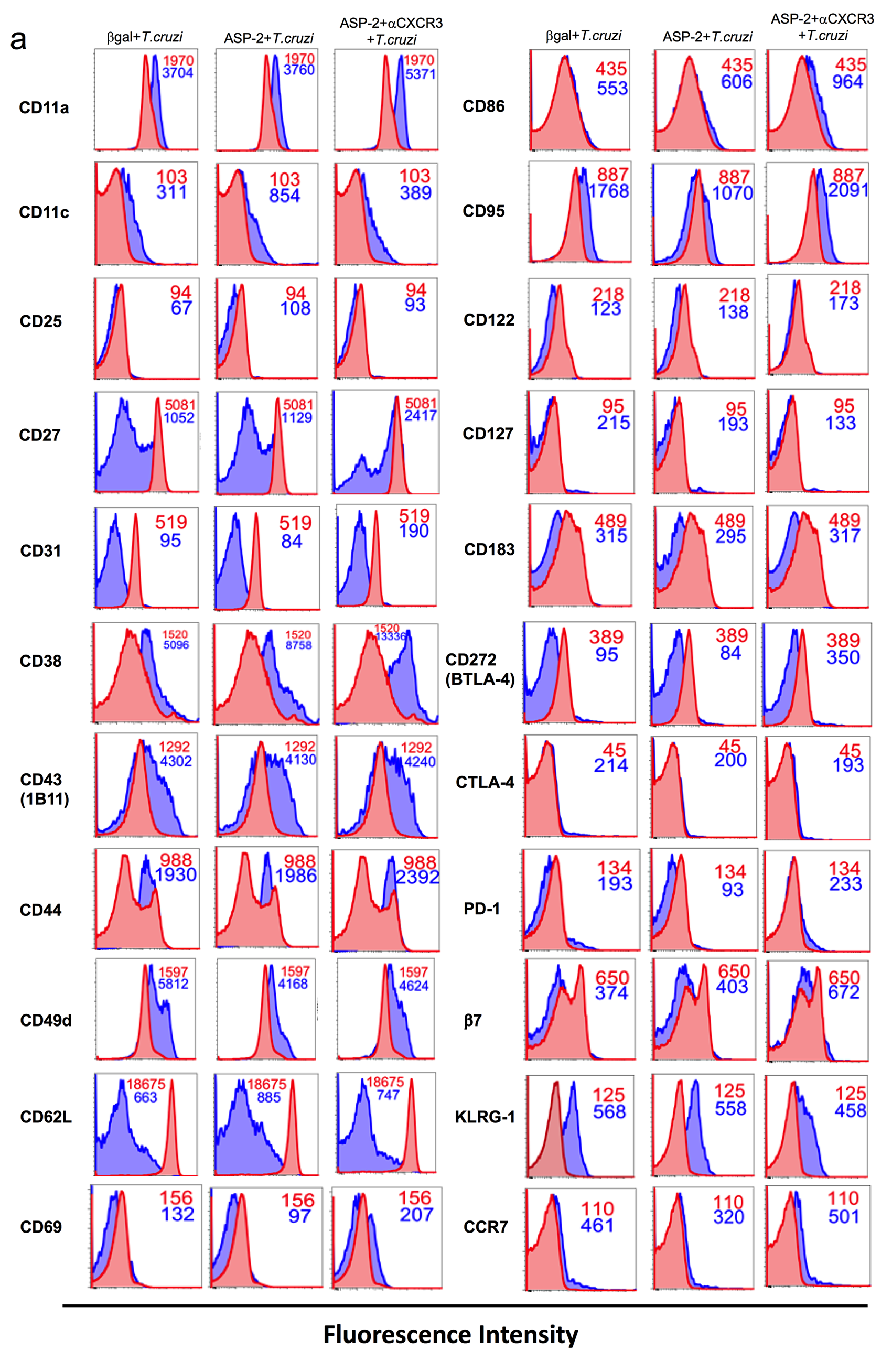

Supplement: S4 Fig — Specific CD8+ T cells were labeled in the spleen using the dextramer H2KK-TEWETGQI with APC-fluorophore and all markers showed in the histograms. a-Histograms represent one animal of each group (βgal+T.cruzi, ASP-2+T.cruzi, and ASP-2+αCXCR3+T.cruzi). The MFI of each marker expressed on the surface of specific CD8+ T cells in the spleen were shown in the histograms. The markers that we choose are related with the activation and stimulation of T lymphocytes. Previously, our group described the effector phenotype on specific CD8+ T cells, as CD44high, CD62Llow and CD11ahigh. The red histogram represents the naïve group and the blue the groups βgal+T.cruzi, ASP-2+T.cruzi, and ASP-2+αCXCR3+T.cruzi. Results are shown as individual values and as the mean ± SEM for each group (n = 4). (TIF) [file pntd.0007597.s004.tif]
